# Tregs facilitate obesity and insulin resistance via a Blimp-1/IL-10 axis

**DOI:** 10.1172/jci.insight.140644

**Published:** 2021-02-08

**Authors:** Lisa Y. Beppu, Raja Gopal Reddy Mooli, Xiaoyao Qu, Giovanni J. Marrero, Christopher A. Finley, Allen N. Fooks, Zackary P. Mullen, Adolfo B. Frias, Ian Sipula, Bingxian Xie, Katherine E. Helfrich, Simon C. Watkins, Amanda C. Poholek, Sadeesh K. Ramakrishnan, Michael J. Jurczak, Louise M. D’Cruz

**Affiliations:** 1 Department of Immunology;; 2 Divison of Endocrinology, Department of Medicine, and the Center for Metabolism and Mitochondrial Medicine;; 3 Center for Biologic Imaging; and; 4 Department of Pediatrics, University of Pittsburgh, Pittsburgh, Pennsylvania, USA.

**Keywords:** Endocrinology, Immunology, Adipose tissue, Cytokines, T cells

## Abstract

Interleukin-10 (IL-10) is a critical cytokine used by immune cells to suppress inflammation. Paradoxically, immune cell–derived IL-10 can drive insulin resistance in obesity by suppressing adipocyte energy expenditure and thermogenesis. However, the source of IL-10 necessary for the suppression of adipocyte thermogenesis is unknown. We show here that CD4^+^Foxp3^+^ regulatory T cells (Tregs) are a substantial source of IL-10 and that Treg-derived IL-10 can suppress adipocyte beiging. Unexpectedly, Treg-specific loss of IL-10 resulted in increased insulin sensitivity and reduced obesity in high-fat diet–fed male mice. Mechanistically, we determined that Treg-specific loss of the transcription factor Blimp-1, a driver of IL-10 expression by Tregs, phenocopied the Treg-specific IL-10–deficient mice. Loss of Blimp-1 expression in Tregs resulted in reduced ST2^+^KLRG1^+^, IL-10-secreting Tregs, particularly in the white adipose tissue. Blimp-1–deficient mice were protected from glucose intolerance, insulin resistance, and diet-induced obesity, through increased white adipose tissue browning. Taken together, our data show that Blimp-1–regulated IL-10 secretion by Tregs represses white adipose tissue beiging to maintain adipose tissue homeostasis.

## Graphical abstract

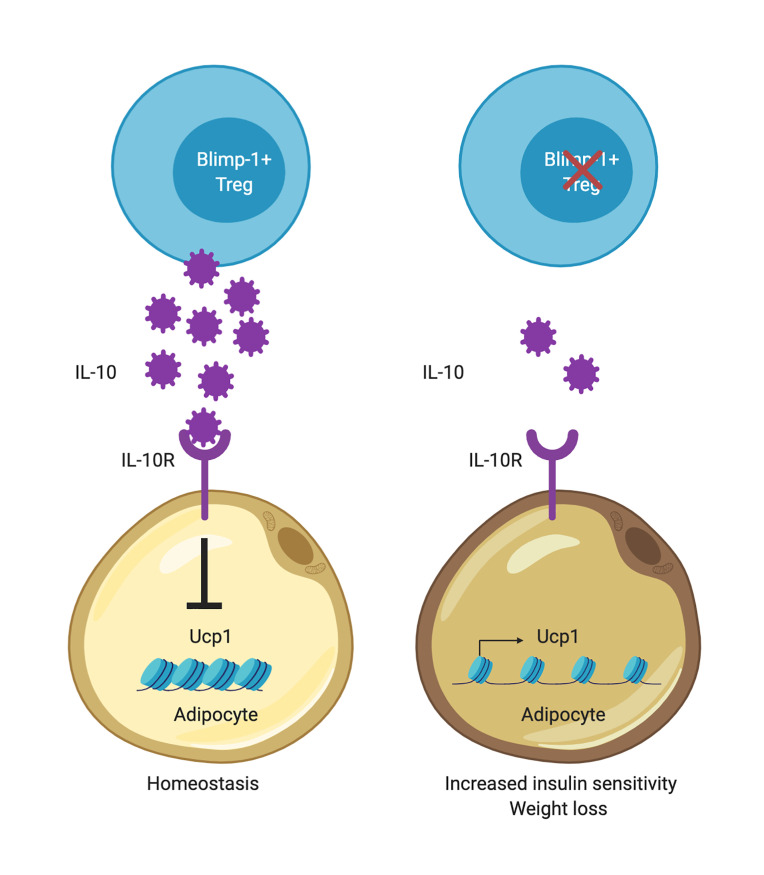

## Introduction

Obesity, an imbalance between energy intake and energy expenditure, is continuing to rise globally at an unchecked pace. Solutions such as lifestyle changes, reduction in high-calorie diet, and increased exercise are difficult to implement and maintain, rendering these solutions largely ineffective as a treatment for obesity. Recently, the possibility of inducing white adipocytes to become “beige” or “brite” has emerged as a potential solution for weight loss and reducing obesity. Upregulation of uncoupling protein 1 (UCP1) in the mitochondria and subsequent uncoupling of the electron transport chain results in beige adipocytes that are adept at dissipating energy as heat (1–3). This increase in energy expenditure by UCP1^+^ beige adipocytes can rebalance energy intake and expenditure, resulting in weight loss (1, 3).

One mechanism for increasing adipocyte beiging and energy expenditure is through immune cell communication with adipocytes (4). Evidence now suggests that cytokines and other mediators immune cells produce are critical for modulating adipocyte beiging. For example, eosinophil-derived IL-4 and innate lymphoid cell–derived (ILC-derived) IL-4 and IL-13 can act on adipocyte precursor cells to initiate differentiation into beige adipocytes (5, 6). Noninflammatory, alternatively activated macrophages have been reported by some groups to secrete norepinephrine to increase adipocyte beiging (6, 7), and ILC2s can produce methionine-enkephalins, opioid-like compounds, to induce beiging in a UCP1-dependent manner (8).

One cytokine with a controversial role in adipocyte–immune cell communication is IL-10. IL-10 is an antiinflammatory cytokine, produced by immune cells and required for the regulation of tissue homeostasis, particularly in the intestine (9). Although previously shown to be required for suppression of adipose tissue inflammation and insulin resistance (10, 11), a recent study showed that IL-10 can directly suppress thermogenesis in adipocytes through a STAT3-dependent signaling pathway (12). Germline deletion of IL-10 protects mice from insulin resistance and diet-induced obesity (DIO), by increasing Ucp1, PR/SET domain 16 (PRDM16), and other thermogenic genes in adipocytes in the white adipose tissue (WAT) (12). Further evidence shows that T and B lymphocytes are the predominant IL-10–producing cells in the adipose tissue (13). Because adipose tissue–resident CD4^+^Foxp3^+^ regulatory T cells (Tregs) have consistently been shown to secrete IL-10 (14, 15), we speculated here that Treg-derived IL-10 could be critical for crosstalk between adipocytes and immune cells to regulate adipose tissue homeostasis.

Our findings reveal that Treg-secreted IL-10, driven by the transcription factor Blimp-1, is critical for suppression of the adipocyte beiging gene program. Treg-specific deletion of IL-10 or Blimp-1 improved insulin sensitivity and DIO. We show here that Treg suppression, through IL-10 secretion, extends beyond communication with autoreactive and inflammatory immune cells and that nonimmune cells, such as adipocytes, are also functionally “suppressed” by Treg-derived IL-10.

## Results

### Treg-specific loss of IL-10 protects mice from DIO.

It was recently reported that IL-10, secreted by immune cells, suppressed adipocyte thermogenesis by repressing expression of UCP1 and other markers associated with adipocyte beiging (12, 13). IL-10 germline deficient mice were significantly protected from DIO, weight gain, and correspondingly, insulin resistance and glucose intolerance (12). Moreover, these findings were not attributable to colitis in the gastrointestinal tracts of these animals (12).

**Figure 1:**
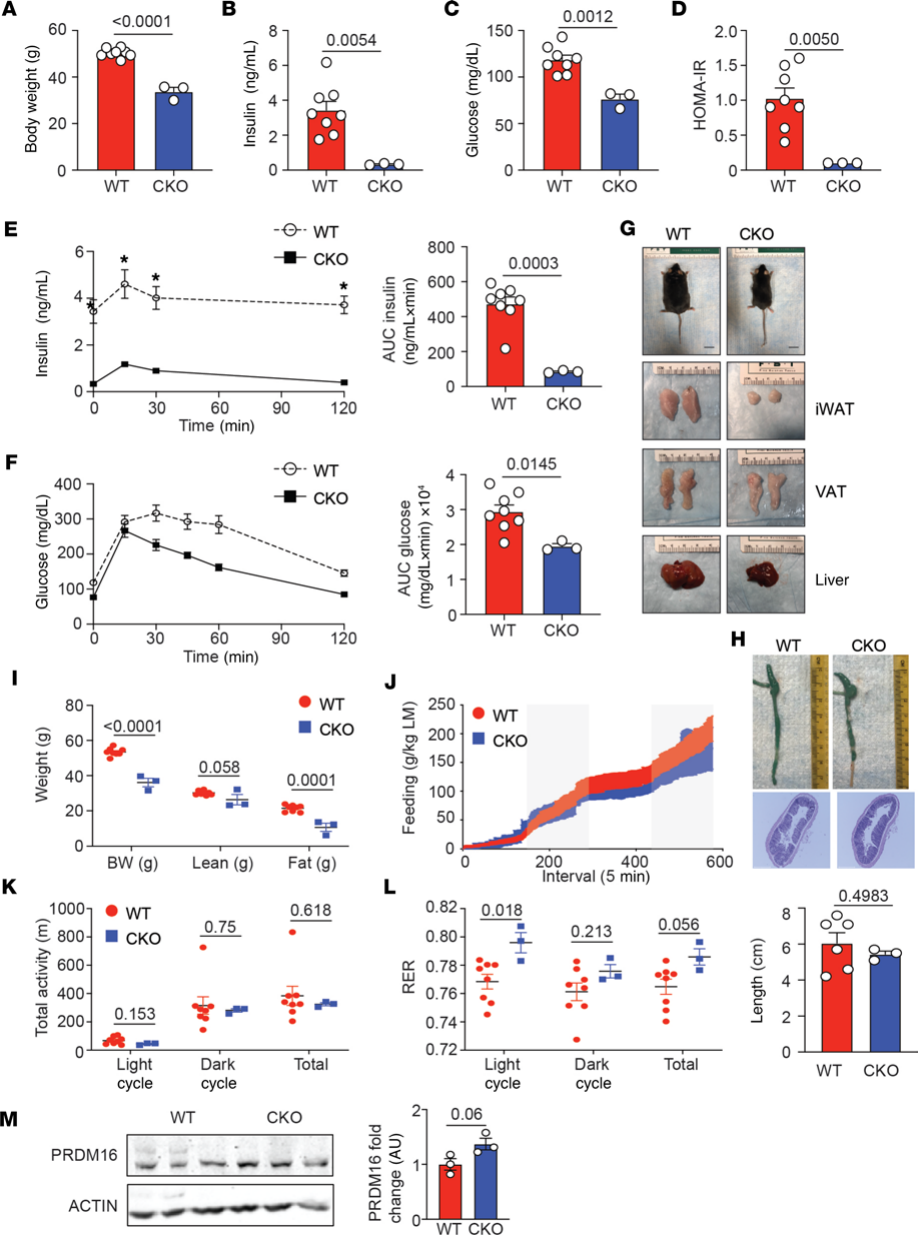
Loss of IL-10 expression by Tregs protects mice from DIO. Male Foxp3-YFP-Cre^+^ (WT) and IL-10^fl/fl^ mice crossed to Foxp3-YFP-Cre^+^ (conditional knockout, CKO) were placed on 60% HFD for 18–20 weeks prior to metabolic analysis. (**A**) Bar graph indicating body weight of 28-week-old HFD-fed WT and CKO mice. (**B**) Bar graph showing fasting plasma insulin levels in WT and CKO mice. (**C**) Bar graph showing fasting blood glucose levels in WT and CKO mice. (**D**) Bar graph showing the homeostatic model assessment of insulin resistance (HOMA-IR) in WT and CKO mice. (**E** and **F**) An i.p. glucose tolerance test (GTT) was performed on WT and CKO mice. The graphs indicate plasma insulin and blood glucose levels in mice over time after i.p. glucose injection. Bar graphs indicate the area under the curve (AUC) for both groups. (**G**) Gross appearance, inguinal WAT (iWAT), epididymal visceral WAT (VAT), and livers of 28-week-old HFD-fed WT and CKO mice. (**H**) Photographs and quantification of the colon length in WT and CKO mice after 18–20 weeks on HFD. Histology showing H&E staining of colon sections from the WT and CKO mice. (**I**) Graph showing body weight (BW) and lean and fat mass in grams of WT and CKO mice as measured by EchoMRI. (**J**–**L**) Food intake in grams per kilogram lean mass (LM), total activity in meters, and respiratory exchange ratio (RER) in light, dark, and total as measured by Promethion Multiplexed Metabolic Cage System during 48-hour total duration. (**M**) Western blots showing PRDM16 expression in total iWAT from WT and CKO mice fed HFD for 18–20 weeks. Each lane represents 1 mouse. β-Actin loading control is shown. Data are presented as means ± SEM and are from 3 independent experiments with 3–8 mice per group, where each dot represents 1 mouse, and an unpaired 2-tailed Student’s *t* test or 1-way ANOVA was performed to determine significance. *P value of less than 0.05.

We first wanted to identify the principal source of IL-10 secreted by immune cells that acted to suppress adipocyte thermogenesis and beiging. It was previously reported that B and T lymphocytes were the primary IL-10 producers in adipose tissue (13). Furthermore, peripheral Tregs, including those residing in the visceral adipose tissue (VAT), were shown to secrete IL-10 (15–17). We thus crossed conditional (*loxp*-flanked) *Il-10* mice to the Foxp3-Cre line to generate IL-10^fl/fl^ Foxp3-Cre^+^ mice to determine if Treg-secreted IL-10 is a primary source of IL-10 required for adipocyte dysregulation and suppression. IL-10^fl/fl^ Foxp3-Cre^+^ mice were placed on 60% high-fat diet (HFD) for 18–20 weeks to induce DIO. Body weight in the IL-10^fl/fl^ Foxp3-Cre^+^ mice was significantly reduced relative to WT littermate controls on HFD (Figure 1A). Consistent with the decrease in body weight, fasting plasma insulin, fasting blood glucose, and insulin resistance (IR) were significantly reduced in IL-10^fl/fl^ Foxp3-Cre^+^ mice compared with WT controls (Figure 1, B–D).

We next performed a glucose tolerance test (GTT) on WT and IL-10^fl/fl^ Foxp3-Cre^+^ mice by injecting glucose i.p. into fasted mice and determining plasma insulin and blood glucose in these animals over a 2-hour period. Although weight differences between cohorts can be a confounding physiological parameter when assessing glucose tolerance, the weight differences between the 2 groups was so marked that it was experimentally unfeasible to test “weight-matched” cohorts. Consistent with our data showing reduced body weight, plasma insulin and blood glucose were substantially reduced in the IL-10^fl/fl^ Foxp3-Cre^+^ mice (Figure 1, E and F).

Because our data phenocopied the IL-10 germline deficient animals on HFD (12), we next determined if the gross morphology of the animals, and their fat pads in particular, were perturbed with Treg-specific loss of IL-10. IL-10^fl/fl^ Foxp3-Cre^+^ mice were smaller in appearance than their WT counterparts (Figure 1G). Furthermore, the iWAT and livers, but not the epididymal VAT, were smaller in the IL-10^fl/fl^ Foxp3-Cre^+^ mice, and the livers appeared smaller and less pale, suggesting protection from hepatic steatosis (Figure 1G).

Treg-secreted IL-10 was previously shown to protect mice from spontaneous colitis (18), and we were concerned that the reduced weight and increased insulin sensitivity in the IL-10^fl/fl^ Foxp3-Cre^+^ mice were due to colitis. However, we observed no difference in colon length with loss of Treg-secreted IL-10, and histology of the WT and IL-10^fl/fl^ Foxp3-Cre^+^ mice was comparable (Figure 1H). Moreover, neither bloody stool nor diarrhea was observed in the IL-10^fl/fl^ Foxp3-Cre^+^ mice (data not shown). We concluded that the reduced weight in the IL-10^fl/fl^ Foxp3-Cre^+^ mice was not due to colitis.

We next performed metabolic analysis of the WT and IL-10^fl/fl^ Foxp3-Cre^+^ mice using the Promethion Multiplexed Metabolic Cage System, as well as body composition measures using ^1^H-NMR. In agreement with the gross morphology data, the fat mass in the IL-10^fl/fl^ Foxp3-Cre^+^ mice was reduced relative to WT mice, while the lean mass in both groups was equal (Figure 1I). While food intake (per kilogram of lean mass [LM]), activity, and energy expenditure were equal between both groups (Figure 1, J and K, and data not shown), the respiratory exchange ratio (RER) was increased in the IL-10^fl/fl^ Foxp3-Cre^+^ mice, relative to the control group, indicating these animals increased whole-body glucose oxidation relative to fat oxidation (Figure 1L).

Increased glucose oxidation is associated with increased energy expenditure and heat output in beige adipocytes (19, 20). Therefore, we next investigated if proteins associated with beige adipocytes were increased in the adipose tissue of IL-10^fl/fl^ Foxp3-Cre^+^ mice. We first measured mRNA expression in iWAT and VAT of IL-10^fl/fl^ Foxp3-Cre^+^ mice. We noted that Ucp1, Prdm16, cell death inducing DFFA like effector a (Cidea), and iodothyronine deiodinase 2 (Dio2), all beige-associated genes, were increased in the IL-10^fl/fl^ Foxp3-Cre^+^ mice, while expression of white-associated genes including adiponectin (AdipoQ), fatty acid binding protein 4 (Fabp4), Perilipin 2 (Plin2), and Pparg were similar between the 2 groups (Supplemental Figure 1; supplemental material available online with this article; https://doi.org/10.1172/jci.insight.140644DS1). At the protein level, we could not detect a significant increase in UCP1 expression (data not shown). However, the transcription factor PRDM16 has been shown to positively regulate UCP1 expression (21), and we could detect a trend in increased PRDM16 protein expression in the adipocytes isolated from the IL-10^fl/fl^ Foxp3-Cre^+^ mice relative to the WT controls (Figure 1M). Our data therefore suggest that Tregs represent a major source of IL-10 that represses beige adipocyte differentiation and thermogenesis.

### Adipose tissue-resident Tregs preferentially express Blimp-1.

**Figure 2:**
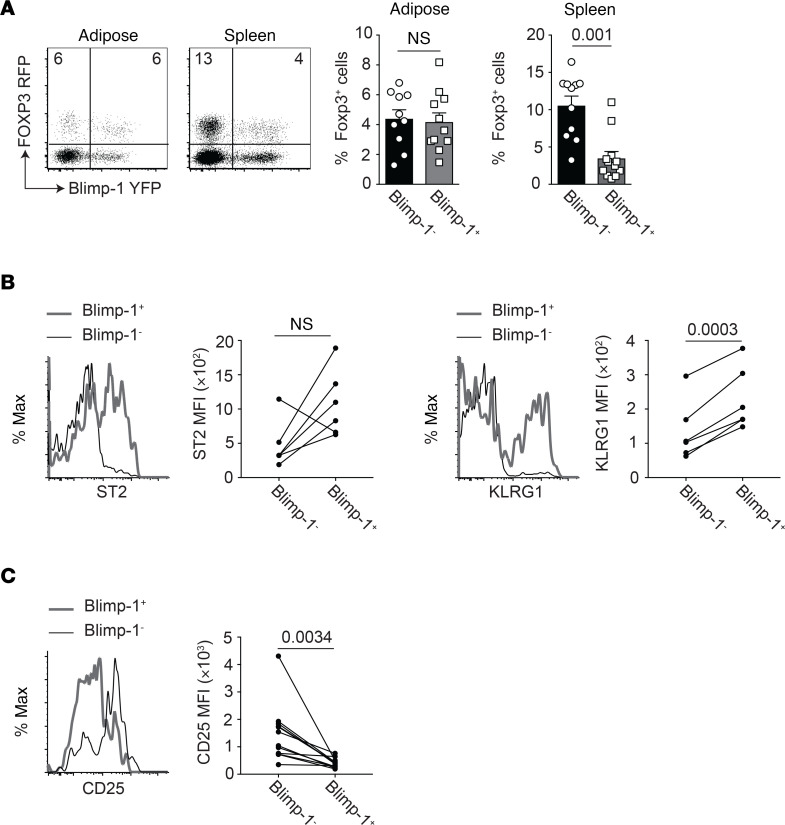
Blimp-1 expression in Tregs from the VAT and spleen. Cells were isolated from the spleen or VAT of 15-week-old male mice expressing Blimp-1–YFP and Foxp3-RFP. (**A**) Flow cytometry plots and bar graphs indicating the frequency of CD4^+^ gated Blimp-1^+^ and Blimp-1^–^Foxp3^+^ Tregs from the indicated tissues. Each dot represents 1 animal. (**B**) Histograms and graphs indicating ST2 and KLRG1 expression on gated CD4^+^Foxp3^+^ aTregs that were either Blimp-1^+^ (gray line) or Blimp-1^–^ (black line). (**C**) Histograms and graphs indicating CD25 expression on gated CD4^+^Foxp3^+^ aTregs that were either Blimp-1^+^ (gray line) or Blimp-1^–^ (black line). Data are presented as means ± SEM for *n* = 11 mice per group (**A**) or *n* = 6 mice per group (**B** and **C**), pooled from 4 independent experiments. A paired 2-tailed Student’s *t* test was performed to determine significance and the *P* values are indicated on the graphs (n.s., not significant).

We next wanted to determine the likely transcriptional regulator of IL-10 secretion from Tregs. The transcription factor Blimp-1 was previously shown to bind to the IL-10 promoter in Tregs and to increase protein expression of IL-10 in these cells (22–24). Previous data have also shown that Blimp-1 expression in Tregs residing in peripheral tissues such as the adipose tissue (aTregs) is high, relative to splenic Tregs, and suggested a correlation between Blimp-1 and the aTreg marker suppression of tumorigenicity 2 (ST2), the IL-33 receptor (15, 17). We first confirmed that Blimp-1 was expressed by aTregs, as previously reported (17). To this end, we crossed Foxp3-RFP mice to Blimp-1–YFP BAC-transgenic reporter mice. In contrast to Tregs in the spleen, we detected an equivalent percentage of Blimp-1^+^ and Blimp-1^–^ Tregs in the VAT (Figure 2A). Our analysis showed that Blimp-1^+^ aTregs expressed increased levels of ST2 and killer cell lectin like receptor G1 (KLRG1), indicating that Blimp-1 expression marked “effector-like” peripheral Tregs in the adipose tissue (Figure 2B). Blimp-1 has been previously reported to repress CD25 expression on CD8^+^ effector T cells (25). Similarly, we noted that CD25 expression was reduced in Blimp-1^+^ aTregs (Figure 2C). Together, these data confirm that a percentage of Tregs in peripheral tissues such as the VAT expressed Blimp-1 and were possible candidates for IL-10 secretion and suppression of adipocyte beiging.

### Loss of Blimp-1 in Tregs results in selective decrease in Tregs in lean, but not obese, mice.

**Figure 3:**
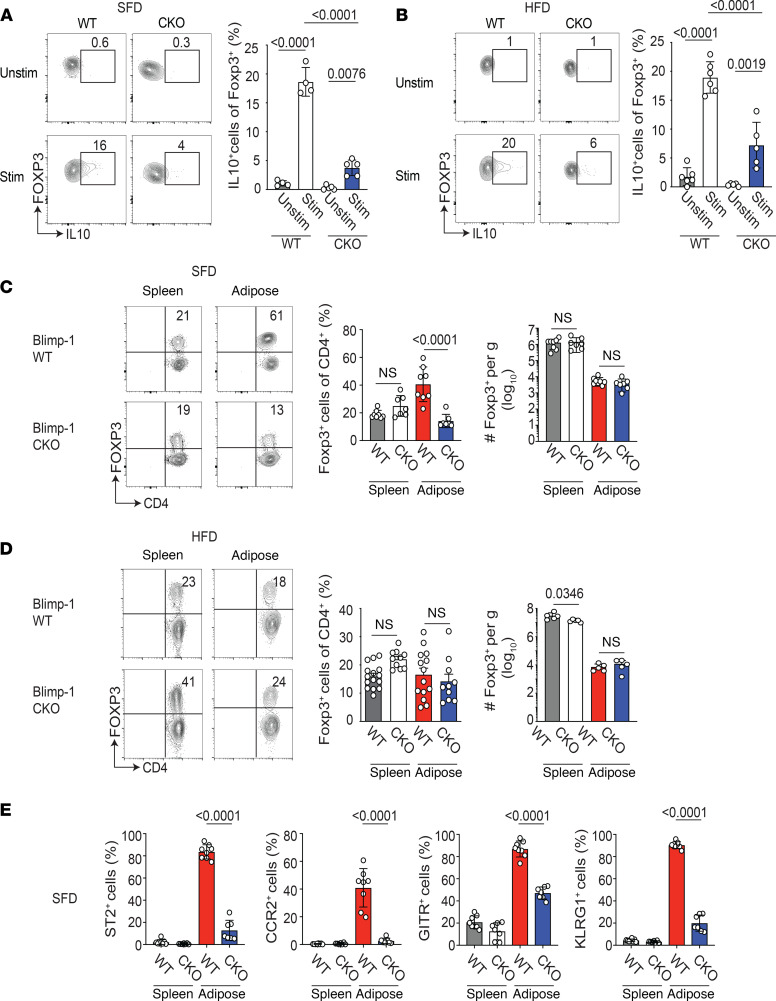
Loss of Blimp-1 expression in VAT Tregs decreases IL-10 secretion and Treg differentiation. Male Foxp3-YFP-Cre^+^ (WT) and Blimp-1^fl/fl^ mice crossed to Foxp3-YFP-Cre^+^ (CKO) were placed on SFD or 60% HFD at 8 weeks of age for 18–20 weeks prior to VAT Treg analysis. (**A**) Flow cytometry plots and bar graphs showing expression of IL-10 by gated CD4^+^Foxp3^+^ cells that were unstimulated (unstim) or stimulated (stim) for 5 hours with PMA and ionomycin from the VAT of SFD-fed WT and CKO mice. (**B**) Flow cytometry plots and bar graphs showing expression of IL-10 by gated CD4^+^Foxp3^+^ cells that were unstimulated (unstim) or stimulated (stim) for 5 hours with PMA and ionomycin from the VAT of HFD-fed WT and CKO mice. (**C**) Flow cytometry plots and bar graphs showing the frequency and number of CD4^+^Foxp3^+^ cells in the indicated tissue from SFD-fed WT and CKO mice. (**D**) Flow cytometry plots and bar graphs showing the frequency and number of CD4^+^Foxp3^+^ cells in the indicated tissue from HFD-fed WT and CKO mice. (**E**) Bar graphs showing expression of ST2, CCR2, GITR, and KLRG1 on gated CD4^+^Foxp3^+^ cells in the indicated tissue from WT and CKO mice on SFD. Each dot represents 1 animal. Data are presented as means ± SEM and are from 2–3 independent experiments with 4–14 mice. An unpaired 2-tailed Student’s *t* test or 1-way ANOVA was performed to determine significance, and the *P* values are indicated on the graphs (n.s., not significant).

Having confirmed that at least half of all Foxp3^+^ Tregs in the VAT express Blimp-1, we next wanted to determine if Treg-specific loss of Blimp-1 would result in reduced IL-10 production by these cells. We crossed transgenic mice expressing Cre recombinase and YFP under the control of Foxp3 regulatory elements to mice carrying conditional (*loxp*-flanked) *Prdm1* alleles to generate Blimp-1^fl/fl^ Foxp3-Cre^+^ mice. We then probed these cells for IL-10 secretion. Using 26- to 28-week-old WT littermate controls or Blimp-1^fl/fl^ Foxp3-Cre^+^ male mice on standard-fat diet (SFD), we isolated Tregs from the VAT and confirmed that IL-10 expression was significantly reduced in Blimp-1–deficient aTregs (Figure 3A). Similarly, when we placed these Blimp-1^fl/fl^ Foxp3-Cre^+^ mice on 60% HFD, aTregs isolated from these animals showed reduced IL-10 expression relative to WT aTregs (Figure 3B). Because IL-10 is secreted by a number of immune cells in the adipose tissue (13), we assessed IL-10 secretion by other T cell populations in the absence of Blimp-1 Treg expression in mice fed SFD and HFD (Supplemental Figure 2). Although IL-10 secretion by CD4^+^ effector T cells was substantial in the VAT, particularly under HFD conditions, we observed few changes in IL-10 secretion by these cells in the absence of Blimp-1 in Tregs (Supplemental Figure 2).

Blimp-1 has also been shown to regulate the differentiation of effector Tregs (15, 17, 22, 23). Thus, we wanted to determine if Blimp-1 deficiency affected other aspects of aTreg biology. Isolating Tregs from the spleen and VAT of 26- to 28-week-old male mice, we determined that the frequency of Tregs was reduced in the visceral adipose tissue but not the spleen under SFD conditions, although the cell numbers per gram of tissue were unaffected (Figure 3C). In HFD-fed mice, the frequency of WT aTregs was reduced relative to WT aTregs from SFD-fed mice, as previously reported (16) (Figure 3, C and D). Blimp-1 deficiency did not affect the frequency or number of Tregs in the spleen or adipose tissue in obese mice (Figure 3D). We next examined expression of ST2, CCR2, TNF receptor superfamily member 18 (GITR), and KLRG1 on Blimp-1–deficient Tregs from SFD-fed mice. Although we observed no difference in expression of these markers in Tregs isolated from the spleen, the frequencies of ST2^+^, CCR2^+^, GITR^+^ and KLRG1^+^ cells were significantly reduced in the Blimp-1–deficient Tregs isolated from the VAT of SFD-fed mice (Supplemental Figure 3A and Figure 3E). Similarly, the ST2^+^, CCR2^+^, GITR^+^, and KLRG1^+^ aTreg frequencies were reduced in the VAT of HFD-fed mice (Supplemental Figure 3B). Together our data show that loss of Blimp-1 expression in Tregs affected expression of surface markers associated with the differentiation of peripheral Tregs, specifically in the VAT.

Tregs reside in other adipose tissue depots (26), so we next examined the frequency and differentiation of Tregs isolated from the iWAT, the inguinal lymph nodes (iLNs), and the brown adipose tissue (BAT) in SFD- and HFD-fed mice. Under SFD conditions, the frequency of Tregs was reduced in the Blimp-1^fl/fl^ Foxp3-Cre^+^ mice relative to the WT mice in the VAT but not in the iWAT or BAT (Supplemental Figure 4A). The frequency of Tregs expressing the differentiation markers ST2 and KLRG1 was decreased in the iWAT and BAT fat depots in a manner similar to what we observed in the VAT (Supplemental Figure 4A). When we placed WT and Blimp-1^fl/fl^ Foxp3-Cre^+^ mice on HFD, we noted that the frequency and phenotype of the Tregs isolated from the iWAT was similar to Tregs isolated from the VAT (Supplemental Figure 4B). Surprisingly, the frequency of Tregs in the BAT increased in Blimp-1^fl/fl^ Foxp3-Cre^+^ mice on HFD but expression of ST2 and KLRG1 was lower in these cells (Supplemental Figure 4B). We also examined IL-10 secretion by stimulated Tregs from the iWAT and iLNs in SFD and HFD-fed mice and showed that Blimp-1 deficiency similarly negatively affects IL-10 secretion from these Tregs (Supplemental Figure 4, C and D). Critically, Blimp-1 deficiency resulted in decreased IL-10 secretion by Tregs in all adipose tissue depots we examined, suggesting that Blimp-1–regulated IL-10 secretion by Tregs could be an essential negative regulator of adipocyte beiging and whole-body metabolism.

### Loss of Blimp-1 in Tregs leads to decreased fat mass and IR.

**Figure 4:**
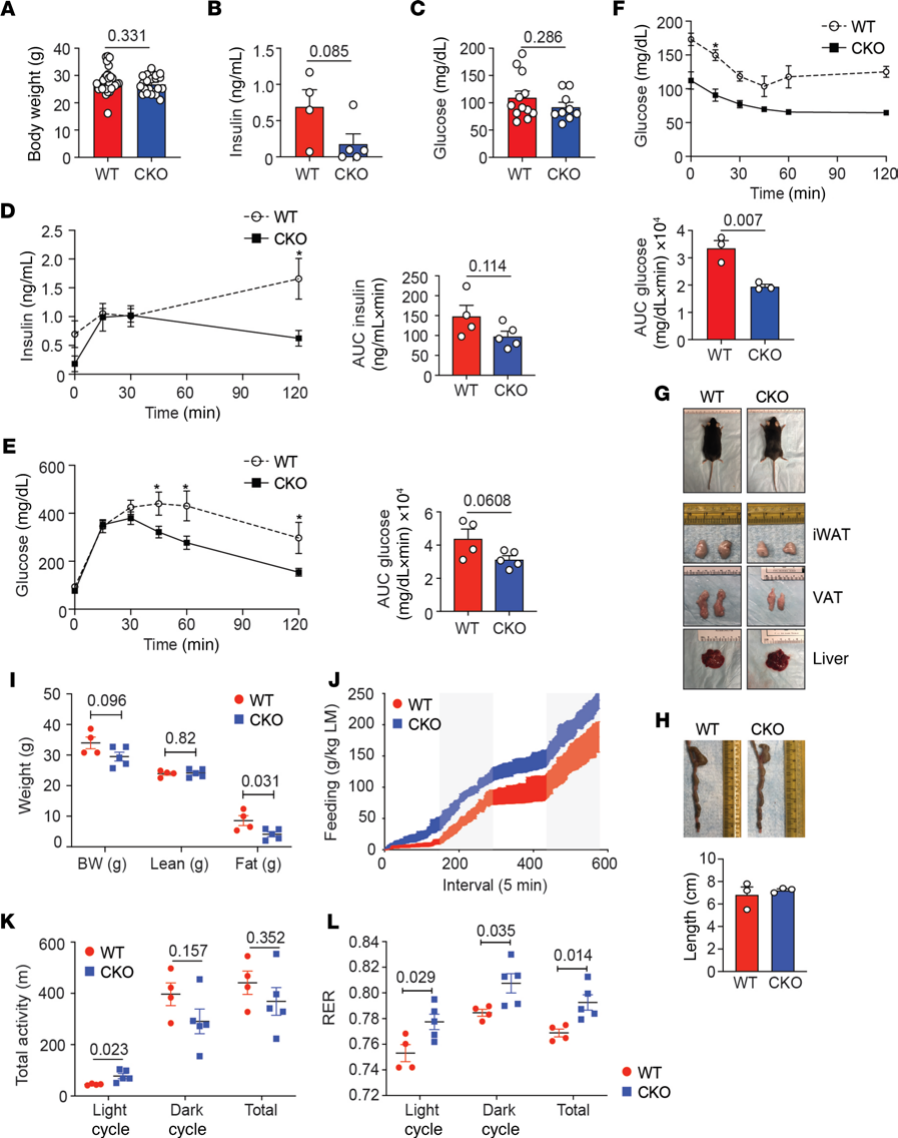
Loss of Blimp-1 expression by Tregs decreases fat mass and increases insulin sensitivity. Male Foxp3-YFP-Cre^+^ (WT) and Blimp-1^fl/fl^ mice crossed to Foxp3-YFP-Cre^+^ (CKO) were placed on SFD and analyzed at 26–28 weeks of age. (**A**) Bar graphs indicating BW of 26- to 28-week-old SFD-fed WT and CKO mice. (**B**) Bar graph showing fasting plasma insulin levels in SFD-fed WT and CKO mice. (**C**) Bar graph showing fasting blood glucose levels in SFD-fed WT and CKO mice. (**D** and **E**) An i.p. GTT was performed on WT and CKO mice. The graphs indicate plasma insulin and blood glucose levels in mice over time after glucose injection. Bar graphs indicate the AUC for both groups. (**F**) An i.p. ITT was performed on WT and CKO mice. The graph indicates blood glucose levels in mice over time after i.p. insulin injection. Bar graph indicates the AUC for both groups. (**G**) Gross appearance, iWAT, epididymal VAT, and livers of SFD-fed WT and CKO mice. (**H**) Photographs and quantification of the colon length in WT and CKO mice. (**I**) Graph showing BW and lean and fat mass in grams of WT and CKO mice as measured by EchoMRI. (**J**–**L**) Food intake in grams per kilogram LM, total activity in meters, and RER in light, dark, and total as measured by Promethion Multiplexed Metabolic Cage System during 48-hour total duration. Data are presented as means ± SEM and are from 2–3 independent experiments with 3–29 mice, where each dot represents 1 mouse, and an unpaired 2-tailed Student’s *t* test or 1-way ANOVA was performed to determine significance. *P value of less than 0.05.

Given our data showing that Blimp-1 deficiency in Tregs isolated from the VAT and iWAT reduced IL-10 secretion from these cells (Figure 3A and Supplemental Figure 4, C and D), we next investigated if body weight and insulin sensitivity were affected in these animals. Body weight was equal between SFD-fed WT and Blimp-1^fl/fl^ Foxp3-Cre^+^animals (Figure 4A). Similarly, fasting serum insulin and blood glucose levels were not significantly different between the 2 groups, although fasting serum insulin trended toward a decrease in the Blimp-1^fl/fl^ Foxp3-Cre^+^ animals (Figure 4, B and C). However, when we performed a GTT by i.p. injecting glucose into overnight fasted mice, we observed that there was a small but significant improvement in glucose tolerance in the Treg-specific Blimp-1–deficient group relative to the WT controls, where plasma glucose levels were significantly less at 45, 60, and 120 minutes after the initial glucose bolus (Figure 4E). Plasma insulin levels were also lower at 120 minutes, and AUC calculations for plasma insulin and glucose levels suggested improved insulin sensitivity and glucose tolerance, respectively (Figure 4, D and E). To further characterize glucose sensitivity in Blimp-1^fl/fl^ Foxp3-Cre^+^ mice, we performed an insulin tolerance test (ITT) by injecting insulin i.p. into 6-hour fasted mice. Consistent with our GTT experiments, we demonstrated that blood glucose levels were significantly reduced in the Blimp-1^fl/fl^ Foxp3-Cre^+^ mice (Figure 4F) in response to insulin.

Examining the gross appearance of the animals on SFD, we could not detect any differences in size between the WT and Blimp-1^fl/fl^ Foxp3-Cre^+^ mice (Figure 4G). However, we did observe that the iWAT and VAT fat pads were slightly smaller in the Blimp-1–deficient animals and that the livers were redder in color, suggestive of reduced hepatic steatosis (Figure 4G). Previous reports have indicated that loss of Blimp-1 expression by Tregs can result in mild intestinal inflammation (24). We did not observe a difference in colon length between WT and Blimp-1^fl/fl^ Foxp3-Cre^+^ animals (Figure 4H), body weight remained the same between the 2 groups (Figure 4A), and we did not observe bloody stool or diarrhea from the Blimp-1^fl/fl^ Foxp3-Cre^+^ animals (data not shown). We concluded that the improved glucose tolerance and insulin sensitivity we observed in the SFD-fed Blimp-1^fl/fl^ Foxp3-Cre^+^ mice was not due to colitis.

In agreement with the gross appearance data (Figure 4G), quantification of body composition by ^1^H-NMR demonstrated that fat tissue mass but not lean tissue mass per gram was reduced in mice with loss of Blimp-1 in Tregs (Figure 4I). Metabolic analysis using the Promethion Multiplexed Metabolic Cage System revealed that despite the reduction in fat tissue mass, Blimp-1^fl/fl^ Foxp3-Cre^+^ mice actually consumed more food per kilogram of LM than their WT counterparts (Figure 4J). Although activity was not different between the 2 groups, the Blimp-1^fl/fl^ Foxp3-Cre^+^ mice did have a higher RER, indicating increased glucose oxidation relative to fat oxidation compared with the WT group (Figure 4, K and L). Taken together, we demonstrate that Blimp-1 expression in aTregs promoted whole-body IR. Further, our data suggest that Blimp-1–regulated IL-10 secretion may be the mechanism by which Tregs regulate insulin sensitivity and whole-body metabolism.

### Treg-specific deletion of Blimp-1 promotes the beige gene program in WAT.

**Figure 5:**
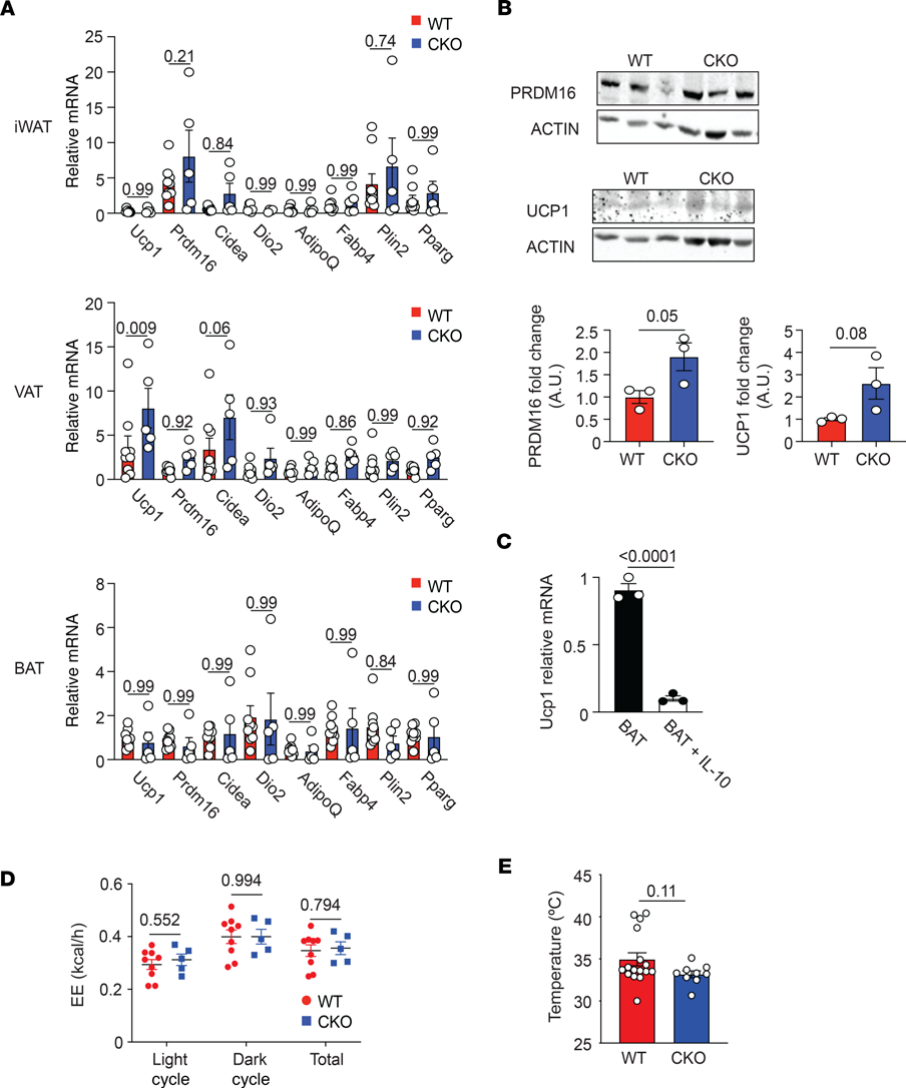
Loss of Blimp-1 expression by Tregs increases adipocyte beiging in WAT. Male Foxp3-YFP-Cre^+^ (WT) and Blimp-1^fl/fl^ mice crossed to Foxp3-YFP-Cre^+^ (CKO) were placed on SFD and analyzed at 26–28 weeks of age. (**A**) Bar graphs showing relative mRNA expression of the indicated gene from total iWAT, VAT, and BAT from 26- to 28-week-old SFD-fed WT and CKO mice. Values were normalized to β-actin. (**B**) Western blots and bar graphs showing UCP1 and PRDM16 expression from total iWAT from SFD-fed WT and CKO mice. Each lane represents 1 biological replicate. β-Actin loading control is shown. (**C**) Graph showing Ucp1 mRNA expression from in vitro differentiated beige adipocyte cell lines and treated with IL-10 for 48 hours. Each dot represents a technical replicate and is representative of 2 independent experiments. (**D**) Graph indicating energy expenditure (EE) in kcal per hour by WT and CKO mice on SFD. (**E**) Bar graph indicating rectal temperature in WT and CKO mice. Data from **A**, **D**, and **E** are presented as means ± SEM and are from 2–3 independent experiments with 3–16 mice, where each dot represents 1 mouse and an unpaired 2-tailed Student’s *t* test or 1-way ANOVA was performed to determine significance.

A recent study has shown that IL-10 suppresses adipocyte function by repressing expression of Ucp1, Prdm16, and other genes associated with adipocyte beiging (12). To determine if adipocyte function was modulated with loss of Blimp-1 expression in Tregs, we isolated whole iWAT and epididymal VAT from WT and Blimp-1^fl/fl^ Foxp3-Cre^+^ mice fed standard chow. In the VAT, mRNA expression of beige tissue–associated genes including Ucp1 and Cidea were significantly increased in the Blimp-1^fl/fl^ Foxp3-Cre^+^ mice relative to WT controls, while expression of WAT-associated genes including AdipoQ, Fabp4, Plin2, and Pparg were unaffected by Blimp-1 deficiency in Tregs (Figure 5A). Despite no significant changes in mRNA beige gene expression in the iWAT, protein expression of UCP1 and PRDM16 were increased in the iWAT of the Blimp-1^fl/fl^ Foxp3-Cre^+^ mice (Figure 5B). We did not detect a significant difference in UCP1 or PRDM16 protein expression in the VAT of Blimp-1^fl/fl^ Foxp3-Cre^+^ mice (data not shown). Together, these data suggested a partial increase in beige gene expression in Blimp-1^fl/fl^ Foxp3-Cre^+^ mice on SFD.

To show that IL-10 can act directly on adipocytes to suppress beiging, we treated in vitro–differentiated brown adipocytes with recombinant IL-10 in vitro. Ucp1 mRNA expression in these cells was dramatically reduced, relative to brown adipocyte cells cultured with vehicle (PBS) (Figure 5C). Despite the increase in beige gene expression by adipocytes in the iWAT and VAT, we did not observe a significant increase in energy expenditure by Blimp-1^fl/fl^ Foxp3-Cre^+^ mice on SFD (Figure 5D). Similarly, consistent with published data (12), we did not observe an increase in body temperature in the Blimp-1^fl/fl^ Foxp3-Cre^+^ mice (Figure 5E).

**Figure 6:**
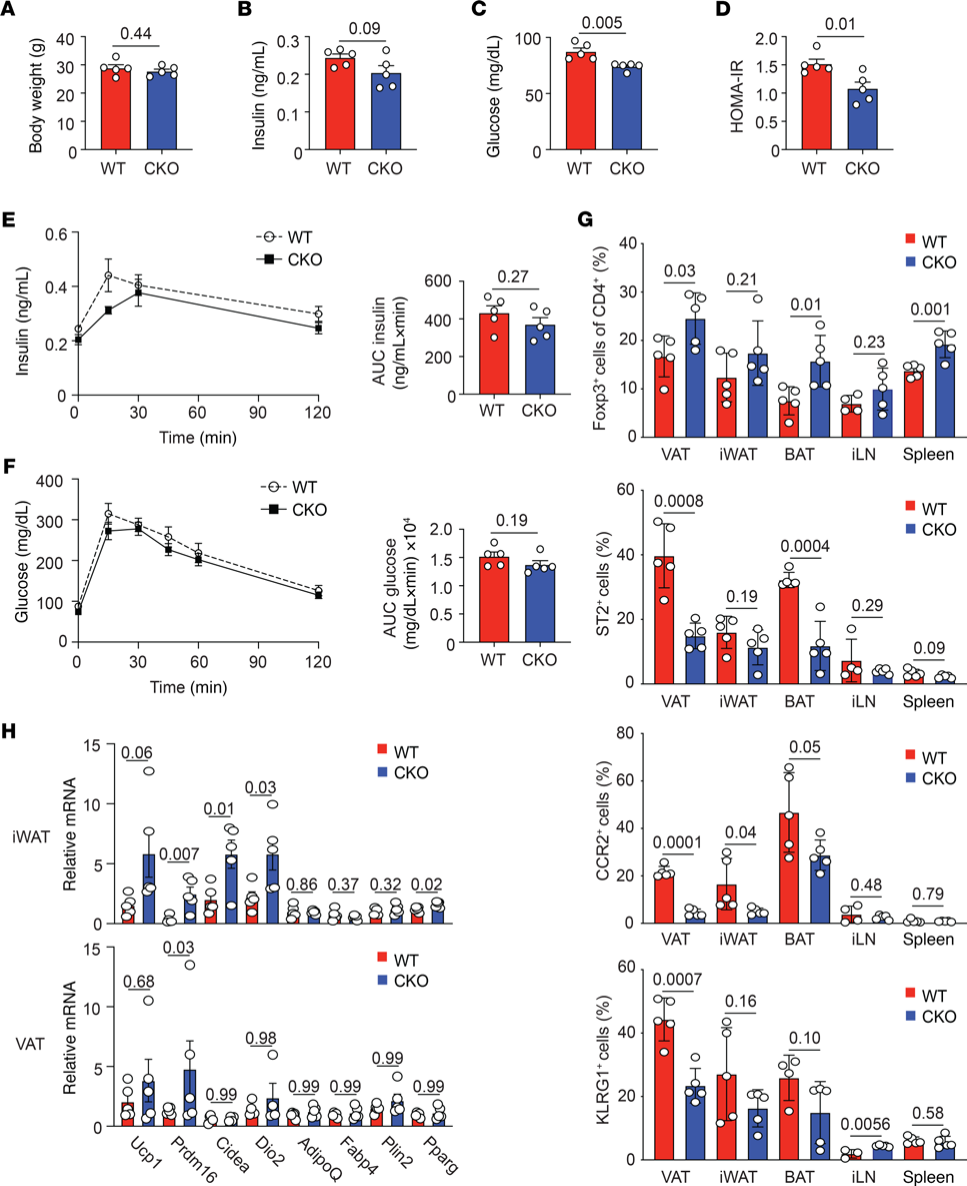
Blimp-1 deficiency on short-term HFD is protective and increases beige gene expression in adipocytes. Eight-week-old male Foxp3-YFP-Cre^+^ (WT) and Blimp-1^fl/fl^ mice crossed to Foxp3-YFP-Cre^+^ (CKO) were placed on 60% HFD for 3 weeks prior to metabolic analysis. (**A**) Bar graphs indicating body weight of 11-week-old HFD-fed WT and CKO mice. (**B**) Bar graph showing fasting plasma insulin levels in WT and CKO mice. (**C**) Bar graph showing fasting blood glucose levels in WT and CKO mice. (**D**) Bar showing the HOMA-IR in WT and CKO mice. (**E** and **F**) An i.p. GTT was performed on WT and CKO mice. The graphs indicate plasma insulin and blood glucose levels in mice over time after glucose injection. Bar graphs indicate the AUC for both groups. (**G**) Bar graphs indicating the percentage of CD4^+^Foxp3^+^ cells in the VAT, iWAT, BAT, inguinal lymph nodes (iLNs), and spleen from short-term HFD-fed WT and CKO mice. Bar graphs also showing expression of ST2, CCR2, and KLRG1 on gated CD4^+^Foxp3^+^ cells in the indicated tissue from WT and CKO mice. (**H**) Bar graph showing relative mRNA expression of the indicated gene from total iWAT and VAT from short-term HFD-fed WT and CKO mice. Values were normalized to β-actin. Each dot represents 1 animal. Data are presented as means ± SEM and are representative of 2 experiments with 2–3 mice per group. An unpaired 2-tailed Student’s *t* test or 1-way ANOVA was performed to determine significance, and the *P* values are indicated on the graphs.

The improvement in insulin sensitivity and glucose tolerance in the Blimp-1^fl/fl^ Foxp3-Cre^+^ mice, and the corresponding increase in adipocyte beige gene expression, were modest in mice on standard chow (Figure 4 and Figure 5). Therefore, we tested the role of Blimp-1 expression and IL-10 secretion by aTregs in mice on HFD for short (3 weeks) and long (18–20 weeks) periods. We first placed 8-week-old male mice on 60% HFD for 3 weeks (short-term HFD). As expected, we observed equal body weight between the 2 groups (Figure 6A). Despite the similarity in body weight, after 3 weeks on HFD, we determined that the fasting plasma insulin, blood glucose levels, and HOMA-IR were significantly decreased in the Blimp-1^fl/fl^ Foxp3-Cre^+^ mice (Figure 6, B–D). We performed a GTT on the short-term HFD-fed WT and Blimp-1^fl/fl^ Foxp3-Cre^+^ mice and observed that plasma insulin and blood glucose levels trended toward improvement in the short-term HFD-fed Blimp-1^fl/fl^ Foxp3-Cre^+^ mice relative to the control group (Figure 6, E and F). Despite the improvement in insulin sensitivity in these animals, we could not determine any changes in body mass, feeding, activity, respiration rate, or energy expenditure in the Blimp-1^fl/fl^ Foxp3-Cre^+^ mice relative to the WT group (Supplemental Figure 5).

We next assessed the phenotype of aTregs isolated from the fat depots of WT and Blimp-1^fl/fl^ Foxp3-Cre^+^ mice on short-term HFD, relative to Tregs from the iLNs and spleen. Surprisingly, loss of Blimp-1 expression in Tregs from these young mice on short-term HFD resulted in an increase in the percentage of aTregs in the VAT and BAT (Figure 6G). However, similar to the aTregs we observed in the fat depots of SFD and HFD-fed 22- to 26-week-old mice (Figure 3), the frequency of ST2^+^, CCR2^+^, and KLRG1^+^ aTregs in the fat depots of young mice were substantially reduced in the absence of Blimp-1 (Figure 6G). Thus, we concluded that Blimp-1 similarly affected Treg differentiation in adipose tissue Tregs, irrespective of the age of the mice or the duration of HFD feeding. Additionally, we investigated IL-10 secretion by aTregs isolated from the VAT and iWAT of the short-term HFD-fed mice. IL-10 secretion was reduced with loss of Blimp-1 expression in Tregs isolated from the VAT, iWAT, iLNs, and spleen (Supplemental Figure 6A), in a manner similar to what we previously observed in the SFD- and HFD-fed groups (Figure 3, A and B).

Finally, we assessed beige gene expression in the adipose tissue depots isolated from WT and Blimp-1^fl/fl^ Foxp3-Cre^+^ mice on short-term HFD. Beige gene expression was substantially increased in the iWAT of Blimp-1^fl/fl^ Foxp3-Cre^+^ mice relative to WT mice, while expression of WAT genes was unchanged between the groups (Figure 6H). Beige gene expression in the VAT was not substantially different in these mice but trended toward increased expression in the Blimp-1^fl/fl^ Foxp3-Cre^+^ mice (Figure 6H). Beige gene expression in the BAT was unperturbed with loss of Treg Blimp-1 expression (Supplemental Figure 6B). Taken together, we concluded that loss of Blimp-1 expression and IL-10 secretion by Tregs was beneficial for improving insulin sensitivity and glucose tolerance through induction of beige gene expression in adipocytes.

### Treg-specific deletion of Blimp-1 protects mice from DIO.

**Figure 7:**
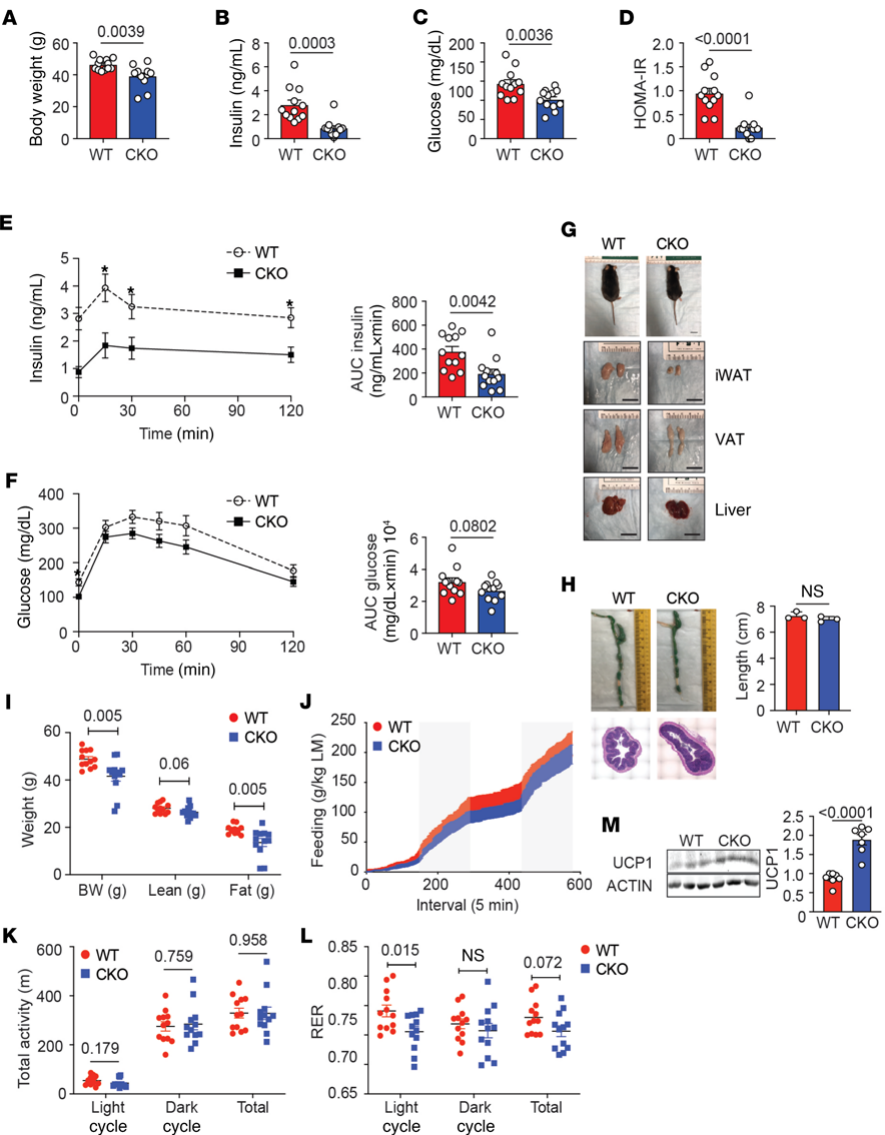
Loss of Blimp-1 expression by Tregs protects mice from DIO. Eight-week-old male Foxp3-YFP-Cre^+^ (WT) and Blimp-1^fl/fl^ mice crossed to Foxp3-YFP-Cre^+^ (CKO) were placed on 60% HFD for 18–20 weeks prior to metabolic analysis. (**A**) Bar graphs indicating body weight of 26- to 28-week-old HFD-fed WT and CKO mice. (**B**) Bar graph showing fasting plasma insulin levels in WT and CKO mice. (**C**) Bar graph showing fasting blood glucose levels in WT and CKO mice. (**D**) Bar graph showing the HOMA-IR in WT and CKO mice. (**E** and **F**) An i.p. GTT was performed on WT and CKO mice. The graphs indicate plasma insulin and blood glucose levels in mice over time after glucose injection. Bar graphs indicate the AUC for both groups. (**G**) Gross appearance, iWAT, epididymal VAT, and livers of 28-week-old HFD-fed WT and CKO mice. (**H**) Photographs and quantification of the colon length in 28-week-old WT and CKO mice after HFD. H&E staining showing cross section of the colon from WT and CKO mice. (**I**) Graph showing BW and lean and fat mass in grams of WT and CKO mice as measured by EchoMRI. (**J**–**L**) Food intake in grams per kilogram LM, total activity in meters, and RER in light, dark, and total as measured by Promethion Multiplexed Metabolic Cage System during 48-hour total duration. (**M**) Western blots and bar graph showing UCP1 expression from total iWAT from 26- to 28-week-old HFD-fed WT and CKO mice. Each lane represents 1 biological replicate. β-Actin loading control is shown. All data are presented as means ± SEM and are from 2–3 independent experiments with 3–12 mice, where each dot represents 1 mouse, and an unpaired 2-tailed Student’s *t* test or 1-way ANOVA was performed to determine significance. *P value of less than 0.05.

As Blimp-1 is a chief regulator of IL-10 secretion by Tregs, we postulated that placing the Blimp-1^fl/fl^ Foxp3-Cre^+^ mice on long-term HFD (18–20 weeks) would recapitulate the metabolic phenotype seen in the HFD-fed IL-10^fl/fl^ Foxp3-Cre^+^ mice (Figure 1). Thus, we placed 8-week-old WT and Blimp-1^fl/fl^ Foxp3-Cre^+^ mice on 60% HFD for 18–20 weeks. We observed that Blimp-1^fl/fl^ Foxp3-Cre^+^ mice had lower body weights, relative to WT controls (Figure 7A). In data comparable with the HFD-fed IL-10^fl/fl^ Foxp3-Cre^+^ mice, the fasting plasma insulin and blood glucose levels were decreased in the Blimp-1^fl/fl^ Foxp3-Cre^+^ mice, and calculation of HOMA-IR, an index of IR, suggested improved insulin sensitivity in these animals, consistent with their protection from DIO (Figure 7, B–D).

We next performed a GTT on the HFD-fed WT and Blimp-1^fl/fl^ Foxp3-Cre^+^ mice by injecting fasted mice with glucose and recording the plasma insulin and blood glucose in these animals for 2 hours after injection. The plasma insulin and blood glucose levels tended toward improvement in the HFD-fed Blimp-1^fl/fl^ Foxp3-Cre^+^ mice relative to the control group, and the AUC calculation for plasma insulin was significantly reduced (Figure 7, E and F). Indeed, analysis of the gross morphology of these animals demonstrated that Blimp-1^fl/fl^ Foxp3-Cre^+^ mice were smaller and that their VAT and iWAT fat pads were smaller (Figure 7G). Similarly, the livers in the HFD-fed Blimp-1^fl/fl^ Foxp3-Cre^+^ mice were smaller and less pale, suggesting reduced steatosis relative to their WT counterparts (Figure 7G). To further characterize glucose sensitivity in the HFD-fed Blimp-1^fl/fl^ Foxp3-Cre^+^ mice, we performed an ITT. Consistent with our GTT experiments, we demonstrated that blood glucose levels trended toward improvement in the Blimp-1^fl/fl^ Foxp3-Cre^+^ mice (Supplemental Figure 8A) in response to insulin.

As with the obese IL-10^fl/fl^ Foxp3-Cre^+^ mice, we were concerned that the phenotype we observed in the obese Blimp-1^fl/fl^ Foxp3-Cre^+^ mice was due to colitis, as Blimp-1 expression in Tregs and local IL-10 production have been associated with suppressing intestinal inflammation (24, 27). However, we detected no differences in the colon length of the WT and Blimp-1^fl/fl^ Foxp3-Cre^+^ mice, colon morphology appeared normal, both groups gained weight over time, and we could detect no bloody stool or diarrhea in the HFD-fed Blimp-1^fl/fl^ Foxp3-Cre^+^ mice (Figure 7H and Supplemental Figure 7, and data not shown).

In agreement with our assessment of the morphology of the fat pads in the Blimp-1^fl/fl^ Foxp3-Cre^+^ mice, when we measured body composition by ^1^H-NMR, we observed that the fat mass per gram in the Blimp-1^fl/fl^ Foxp3-Cre^+^ mice was reduced while the LM remained equal between the 2 groups (Figure 7I). One possibility for the weight differences between the 2 groups was that Blimp-1^fl/fl^ Foxp3-Cre^+^ mice simply ate less than their WT counterparts. However, both groups appeared to eat at the same rate and displayed no differences in activity, indicating that food intake per kilogram LM and activity could not account for the weight differences between the groups (Figure 7, J and K). Analysis of the RER demonstrated that Blimp-1^fl/fl^ Foxp3-Cre^+^ mice had reduced glucose oxidation relative to fat oxidation during the light cycle (Figure 7L).

We next determined if increased adipocyte beiging in obese Blimp-1^fl/fl^ Foxp3-Cre^+^ mice could account for their reduced body weight and increased insulin sensitivity, similar to what we had observed in the SFD mice and in mice on HFD for 3 weeks. We examined expression of Ucp1, Prdm16, Cidea, and Dio2, genes associated with adipocyte beiging, and observed that Ucp1, Prdm16, and Dio2 mRNA expression in particular were increased in the iWAT and VAT of Blimp-1^fl/fl^ Foxp3-Cre^+^ mice (Supplemental Figure 8B). Expression of beige and white genes in the BAT was unaffected with Treg-specific Blimp-1 deficiency (Supplemental Figure 8B). Similarly, Western blot analysis of whole iWAT indicated that UCP1 protein expression was significantly increased in the Blimp-1^fl/fl^ Foxp3-Cre^+^ mice relative to WT controls (Figure 7M). Although energy expenditure between the 2 groups of mice was not significantly different (Supplemental Figure 8C), we did observe an increase in body temperature in Blimp-1^fl/fl^ Foxp3-Cre^+^ mice relative to WT mice (Supplemental Figure 8D), indicating an increase in heat production in these animals.

Finally, we determined if there were significant changes to other immune cell populations in the Blimp-1^fl/fl^ Foxp3-Cre^+^ mice on HFD. We did not observe a difference in the frequency of total CD45^+^ cells from the stromal vascular fraction (SVF) or indeed a difference in the frequency of aTreg^–^ST2^+^CD45^+^ cells in the Blimp-1^fl/fl^ Foxp3-Cre^+^ mice relative to WT mice (Supplemental Figure 9A), leading us to conclude that the increased insulin sensitivity and weight loss we observed in Blimp-1^fl/fl^ Foxp3-Cre^+^ mice was not due to expansion of other ST2^+^ immunoregulatory cells in the VAT and rather due to increased beige gene expression by adipocytes. Because loss of Blimp-1 expression in aTregs in HFD-fed mice perturbed aTreg differentiation and resulted in protection of these animals from DIO, we next tested if other immune cell populations had also been affected by loss of Blimp-1 expression in the aTregs. However, the frequencies of macrophage subsets, B cells, and DCs were all unaffected by loss of Blimp-1 expression in aTregs (Supplemental Figure 9, B and C). Taken together, our data strongly support our hypothesis that it is Treg-secreted IL-10, driven by Blimp-1 expression, that suppresses adipocyte beiging and potentiates IR in obesity.

## Discussion

The role of Tregs in peripheral tissues has been extensively explored over the past decade, with most publications delineating a role for these cells in suppressing local inflammation and maintaining tissue homeostasis (14–17, 28–33). However, recent publications have revealed that the function of peripheral Tregs is more complex than initially reported and is tissue and context dependent. For example, aTregs have been shown to be pathogenic in age-associated obesity (34, 35). In addition, WT female mice, which have reduced aTreg frequency and IL-10 secretion, are protected from glucose intolerance and fat gain, relative to age-matched male mice, although whether glucose tolerance and fat reduction in female mice are driven through an IL-10–deficient aTreg axis has not yet been established (15). Similarly, the role of Blimp-1 in the immune system is highly context dependent and tissue specific. For example, it was recently shown that Blimp-1 is an unexpected positive regulator of Th2 responses in the lung, facilitated through an IL-10/STAT3 axis (36).

Treg-secreted IL-10 is known to suppress inflammatory immune cells and for the maintenance of tissue homeostasis, particularly at barrier surfaces (9, 37). In this present study, we show that Treg IL-10 is also critical for suppressing adipocyte beiging in male mice. Loss of IL-10 expression by Tregs resulted in mice that were protected from DIO and showed improved insulin sensitivity. Mechanistically, Treg-intrinsic loss of Blimp-1 led to reduced IL-10 production, resulting in increased expression of PRDM16 and UCP1 by adipocytes, proteins that are critical for upregulation of the beiging program (12, 21, 38). One puzzling aspect of our findings was that whole-body energy expenditure did not increase with loss of IL-10 or Blimp-1 by Tregs, despite an increase in beige gene expression in the visceral and inguinal adipose tissue depots. IL-10 is, however, secreted by other B and T cell populations in the adipose tissue (13), and it may be that IL-10 from these additional cells counters an increase in whole-body expenditure in our model.

It is well established that IL-10 secretion by Tregs is essential for preventing gut inflammation and colitis (18, 24, 27). However, in our system, we found no evidence of Treg-specific deletion of IL-10 or Blimp-1 resulting in colitis and influencing the weight loss phenotype we observed. Mice on HFD gained weight, albeit at a slower rate than their WT counterparts. Possible factors for these distinctions may include the animal’s microbiota and the variety of chow used across studies, as well as the age and subtle differences in the background of the mice. We were encouraged to see our phenotype was consistent with that of the recently published germline IL-10–deficient animals (12) and that colitis was likely not a factor underlying the weight loss and IR we observed in the Treg-specific IL-10– and Blimp-1–deficient animals.

Finally, we consider our findings in the context of previously reported aTreg functions in DIO. Prior data have shown a clear correlation between reduced aTreg frequency and increased body mass and that temporal ablation of Tregs increases IR and adipose tissue inflammation (16). In our study, we report similar reductions in aTreg frequency in both WT and Blimp-1–deficient mice when these animals were placed on HFD. However, our data uniquely demonstrated that disruption of aTreg IL-10 secretion can reduce IR. Excluding IL-10, Tregs can secrete a number of other antiinflammatory soluble mediators, including TGF-β, IL-35, amphiregulin, and methionine-enkephalin (39–42). Hence, our findings suggest that the sum total of these Treg-derived mediators may need to be considered to accurately define how Tregs dictate whole-body metabolism and adipose tissue inflammation.

Together, our findings reveal that IL-10 secretion by Tregs is not limited solely to suppression of effector and autoreactive immune cells (9, 37), but rather that Tregs release IL-10 to promote the suppression of the beige gene program in adipocytes. Ultimately, these data have implications for the role of the immune cell/adipocyte axis in mediating systemic metabolism and protection from DIO.

## Methods

### Study design.

The objective of this study was to interrogate the function of IL-10 and Blimp-1 expression by adipose-resident Tregs in mice on SFD and HFD. To do this, we used a combination of in vivo and ex vivo assays on mouse samples. We designed and performed the experiments predominantly using flow cytometry and metabolic indices for our analysis. The number of replicates for each experiment is indicated in the figure legends.

### Mice.

C57BL/6 mice were purchased from The Jackson Laboratory. Blimp-1–YFP (commercially available at Jackson, stock number 008828), Foxp3-RFP (commercially available at Jackson, stock number 008374) (43), and Blimp-1^fl/fl^ mice (commercially available at Jackson, stock number 008100) (44) were donated by A. Poholek (University of Pittsburgh). IL-10^fl/fl^ mice were donated by D. Vignali (University of Pittsburgh). Foxp3-YFP-Cre mice were purchased from The Jackson Laboratory (stock number 016959) (18). All experiments were approved by the University of Pittsburgh Institutional Animal Care and Use Committee. All mice used in experiments were age-matched littermate males between 26 and 28 weeks of age. Male mice were used due to the hormonal fluctuations that occur in female mice. Where indicated, mice were fed either SFD (standard chow) or HFD (60% kcal fat, Teklad, Envigo, TD06414) from 8 weeks of age until 26–28 weeks of age. Male animals were assigned to groups of 3–7 mice per experiment where possible, and at least 2 independent experiments were performed throughout the study. Mice were bred and housed in specific pathogen–free conditions in accordance with the Institutional Animal Care and Use Guidelines of the University of Pittsburgh. Mice were maintained at approximately 22°C room temperature as standard.

### Cell isolation.

Murine epididymal, inguinal, and brown adipose single-cell suspensions were prepared as previously described (45). Briefly, tissue was harvested, weighed, finely chopped with a razor blade, and digested with Liberase TM and DNase I (MilliporeSigma) at 37°C with shaking for 30 minutes before filtering through a 70 μm nylon mesh and centrifugation for 5 minutes at 4°C. The supernatant was removed and the SVF was isolated and processed for flow cytometry. Single-cell suspensions of spleen and iLN cells were made by processing murine spleens between 2 glass slides.

### Flow cytometry.

Antibodies against murine CD4 (RM4-5, AF700, BV650), CD8 (53-6.7, APC/Fire 750, BV785), CCR2 (SA203G11, BV421), CD11c (N418, APCCy7, Pe/Cy5), CD206 (C068C2, APC), CD11b (M1/70, BV421), F4/80 (BM8, BV510, BV711, FITC), TCRβ (H57-597, BV605), and NK1.1 (PK136, BV650) were purchased from BioLegend. Antibodies against CD4 (GK1.5, BUV395) and ST2 (U29-93, BV480) were purchased from BD Biosciences. Antibodies against B220 (RA3-6B2, AF488), CD11c (N418, e450, PE-Cy5.5), CD25 (PC61.5, PE-Cy7), CD4 (GK1.5, APC-eFluor 780, Super Bright 645), CD45 (30-F11, Pacific Orange), Foxp3 (FJK-16s, APC, e450, FITC), GITR (DTA-1, Super Bright 600), IL-10 (JES5-16E3, PE), KLRG1 (2F1, APC, APC-eFluor 780, PE-eFluor 610), NK1.1 (PK136, PE), and ST2 (RMST2-2, PE, PerCP-eFluor710) were purchased from eBioscience, Thermo Fisher Scientific. Viability dyes (e506, near IR, UV) were purchased from Invitrogen, Thermo Fisher Scientific.

Extracellular flow staining was performed in 2% FBS-PBS in the presence of BioLegend’s TruStain FcX for 30 minutes at 4°C. Fixation, permeabilization, and intracellular staining were performed using eBioscience’s (Thermo Fisher Scientific) Foxp3/transcription factor staining buffer set per manufacturer’s directions. Flow data were collected using a Cytek Aurora or BD Biosciences LSR and analyzed using FlowJo.

### Intracellular cytokine analysis.

For intracellular cytokine staining total SVF was stimulated with PMA (50 ng/mL) (MilliporeSigma) and ionomycin (1 nM) (MilliporeSigma) for 5 hours. Brefeldin A (eBioscience, Thermo Fisher Scientific) was added to the culture at the recommended concentration. Cells were surfaced stained, before fixing, permeabilizing, and intracellular staining according to the manufacturer’s instructions (eBioscience, Thermo Fisher Scientific, Foxp3/transcription factor staining kit).

### Determination of fasting blood glucose, glucose tolerance testing (GTT), and insulin tolerance testing (ITT).

Mice were fasted overnight before fasting blood glucose and insulin levels were measured. Blood glucose was measured using a handheld glucometer (Bayer Contour Next), and plasma insulin was measured by ELISA (Alpco). For the GTTs, we administered glucose (2 g/kg of body weight) by i.p. injection after an overnight fast. We measured changes in plasma insulin at 15, 30, and 120 minutes and blood glucose at 15, 30, 45, 60, and 120 minutes after glucose injection. For the ITT, mice were fasted for 6 hours before intravenous injection with 0.75 U/kg of body weight Novolin-R human recombinant insulin (Henry Schein). After insulin injection, blood glucose was measured at 15, 30, 45, 60, and 120 minutes.

### Indirect calorimetry and body composition measurements.

Indirect calorimetry was performed using the Promethion Multiplexed Metabolic Cage System (Sable Systems) by the University of Pittsburgh Center for Metabolism and Mitochondrial Medicine. Animals were placed individually in chambers for 24 hours to acclimate followed by 48 hours at ambient room temperature with 12-hour light/12-hour dark cycles for analysis. Animals had free access to food and water. Respiratory measurements (VO_2_/VCO_2_) were made at 5-minute intervals. Food intake was measured in metabolic chambers during the 48-hour period. Body composition including fat and LM was determined using by EchoMRI. Mice were weighed twice per month to determine weight increases over time on SFD and HFD.

### Tissue H&E staining.

Tissues were placed in cassettes and submerged in 10% formalin solution overnight. Tissue cassettes were washed with tap water for 15 minutes and stored in 70% ethanol at room temperature. Paraffin embedment and sectioning (4–5 μm thickness) was performed by StageBio. Slides were deparaffinized and rehydrated using ethanol and PBS, respectively. H&E staining was performed using hematoxylin for 5 minutes followed by eosin for 15 seconds. The images were acquired by using an Evos fluorescence inverted microscope.

### Reverse transcription quantitative PCR analysis.

Total RNA was extracted using RNeasy Lipid Tissue Mini Kit (Qiagen) as per the manufacturer’s instructions. First 1 μg of total RNA was reversed-transcribed using Mu-MLV reverse transcriptase (Promega). Gene expression was evaluated by quantitative real-time PCR using SYBR Green Mastermix (Radiant Molecular Tools) with a Quant Studio 3 Station quantitative PCR machine (Applied Biosystems, Thermo Fisher Scientific). The results were expressed as fold change using the 2^-ΔΔCT^ method, and β-actin was used as an internal normalization control. A list of the primer sequences used is provided in Supplemental Table 1.

### Western blotting.

Protein extracts were prepared as previously described (46). In brief, the adipose tissue samples were lysed in radioimmunoprecipitation assay lysis buffer (50 mM Tris pH 7.4, 150 mM NaCl, 5 mM EDTA, 1% NP-40) supplemented with protease and phosphatase inhibitors (MilliporeSigma). The protein content was measured by using protein assay kit (Bio-Rad). The protein lysates (20–25 μg) were resolved on 9%–12% polyacrylamide gels and transferred to nitrocellulose membranes. Membranes were blocked with 5% skim milk powder in Tris-buffered saline containing 0.01% Tween 20 and probed with primary antibodies (UCP1, PAI-24894, Invitrogen, Thermo Fisher Scientific; PRDM16, NBP1-77096, Novus Biologicals; β-actin, 66009-I-Ig, Proteintech) overnight at 4°C. Membranes were then probed with respective secondary antibodies — anti-rabbit IgG (H+L) DyLight800, Cell Signaling Technology, catalog 5151S, and anti-mouse IgG (H+L) DyLight680, Cell Signaling Technology, catalog 5470S) and visualized using Odyssey CLx Imaging System (LI-COR). The Western blot images were quantified by using ImageJ software (NIH, Bethesda, Maryland, USA).

### Cell culture and IL-10 in vitro assay.

Mouse inguinal adipose-derived SVF preadipocytes (Kerafast, Inc.) were provided in-house (University of Pittsburgh). Cells were grown to confluence in DMEM/F12 with 10% FBS and 1% penicillin-streptomycin. One day prior to differentiation, cells were subcultured into a 12-well plate at 70% confluence and allowed to rest overnight. The cells were differentiated into brown adipocytes with the cocktail of 0.5 mM isobutylmethylxanthine, 125 nM indomethacin, 2 μg/mL dexamethasone, 850 nM insulin, 1 nM T3, and 0.5 μM rosiglitazone. After 2 days, cells were placed into postdifferentiation maintenance media consisting of complete media with 850 nM insulin, 1 nM T3, and 0.5 μM rosiglitazone. After an additional 2 days, cells were treated for 16 hours with 100 ng/mL recombinant mouse IL-10 (R&D Systems, Bio-Techne) for downstream real-time PCR analysis.

### Statistics.

All graphs were created using GraphPad Prism 8, and statistical significance was determined with the 2-tailed paired or unpaired Student’s *t* test or using 1-way ANOVA adjusted for multiple comparisons where appropriate. We used a *P* value of less than 0.05 as statistically significant. The graphical abstract was created using BioRender.com.

### Study approval.

All rodent breeding and experimental procedures were approved by and performed in accordance with the guidelines of the Institutional Animal Care and Use Committee of the University of Pittsburgh and complied with the NIH *Guide for the Care and Use of Laboratory Animals* (National Academies Press, 2011).

## Author contributions

LYB conducted the experiments, analyzed the data, and wrote the manuscript; XQ, GJM, CAF, ANF, ZPM, and ABF conducted the experiments; IS, BX, and MJJ performed the metabolic studies and analysis; RGRM and SKR performed the mRNA quantitative PCRs, Western blots, and analysis on adipose tissue; KEH. and SCW advised with microscopy analysis; ACP provided scientific insight; and LMD designed the experiments, analyzed the data, and wrote the paper.

## Supplementary Material

Supplemental data

## Version 1 12/22/2020

In-Press Preview

## Version 2 02/08/2021

Electronic publication

## Version 3 04/20/2021

Corrected author name
