# Supplementary material for: Tregs facilitate obesity and insulin resistance via a Blimp-1/IL-10 axis
Source: JCI Insight. 2021 Feb 8;6(3):e140644. doi: 10.1172/jci.insight.140644 (PMC7934851; doi:10.1172/jci.insight.140644)
Supplement: Supplemental data [file jciinsight-6-140644-s008.pdf]

Supplemental Figure 1

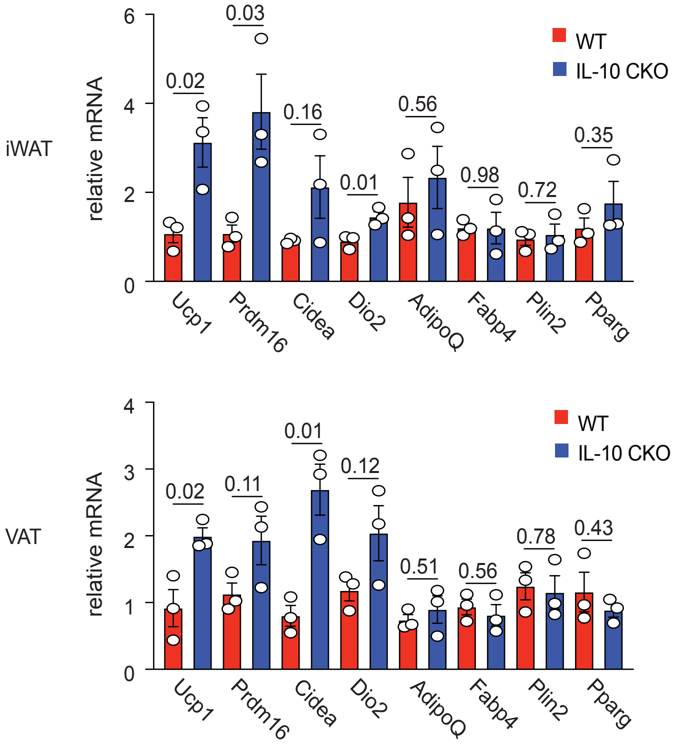

Supplemental Figure 1. Beige and white gene expression in iWAT and VAT adipocytes from Treg specific IL-10 deficient mice. 8-week-old male Foxp3-YFP-Cre+ (WT) and IL-10f/f Foxp3-YFP-Cre+ mice (conditional knockout, IL-10 CKO) were placed on 60% high fat diet (HFD) for 18-20 weeks prior to metabolic analysis. Bar graphs showing relative mRNA expression of the indicated gene from total iWAT and VAT from 26-28-week-old HFD-fed WT and IL-10 CKO mice. Values were normalized to beta-actin. Data are presented as means ± s.e.m. for n = 3 mice per group. An unpaired Student’s t-test was performed to determine significance and the P values are indicated on the graphs.

Supplemental Figure 2

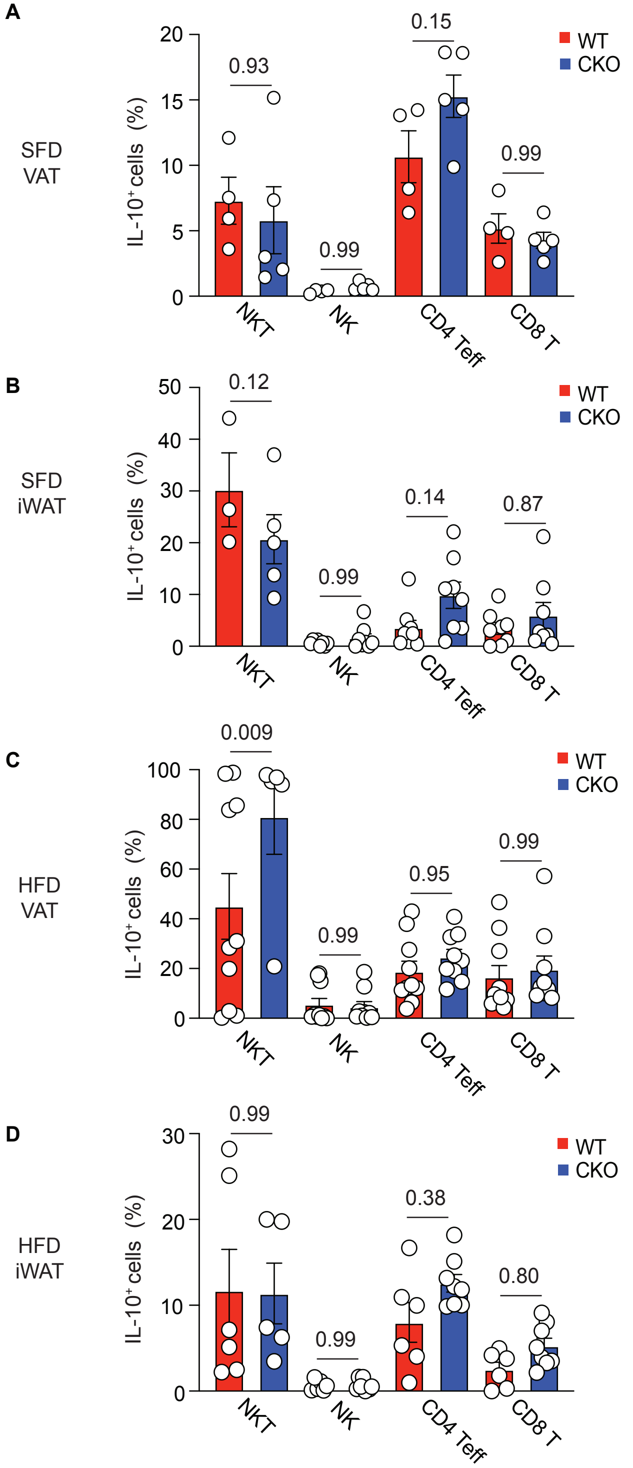

Supplemental Figure 2. IL-10 secretion by other immune cells in the VAT and iWAT. Male Foxp3-YFP-Cre+ (WT) and Blimp-1f/f mice crossed to Foxp3-YFP-Cre+ (conditional knockout, CKO) were placed on standard fat diet (SFD) or 60% high fat diet (HFD) at 8 weeks of age for 18-20 weeks prior to analysis. Bar graphs indicating the percentage of IL-10+ immune cells gated on live CD45+ lymphocytes isolated from (A-B) SFD VAT and iWAT and (C-D) HFD VAT and iWAT. Data are presented as means ± s.e.m. for n = 3-8 mice per group pooled from two independent experiments. An unpaired Student's t-test was performed to determine significance and the P values are indicated on the graphs.

Supplemental Figure 3

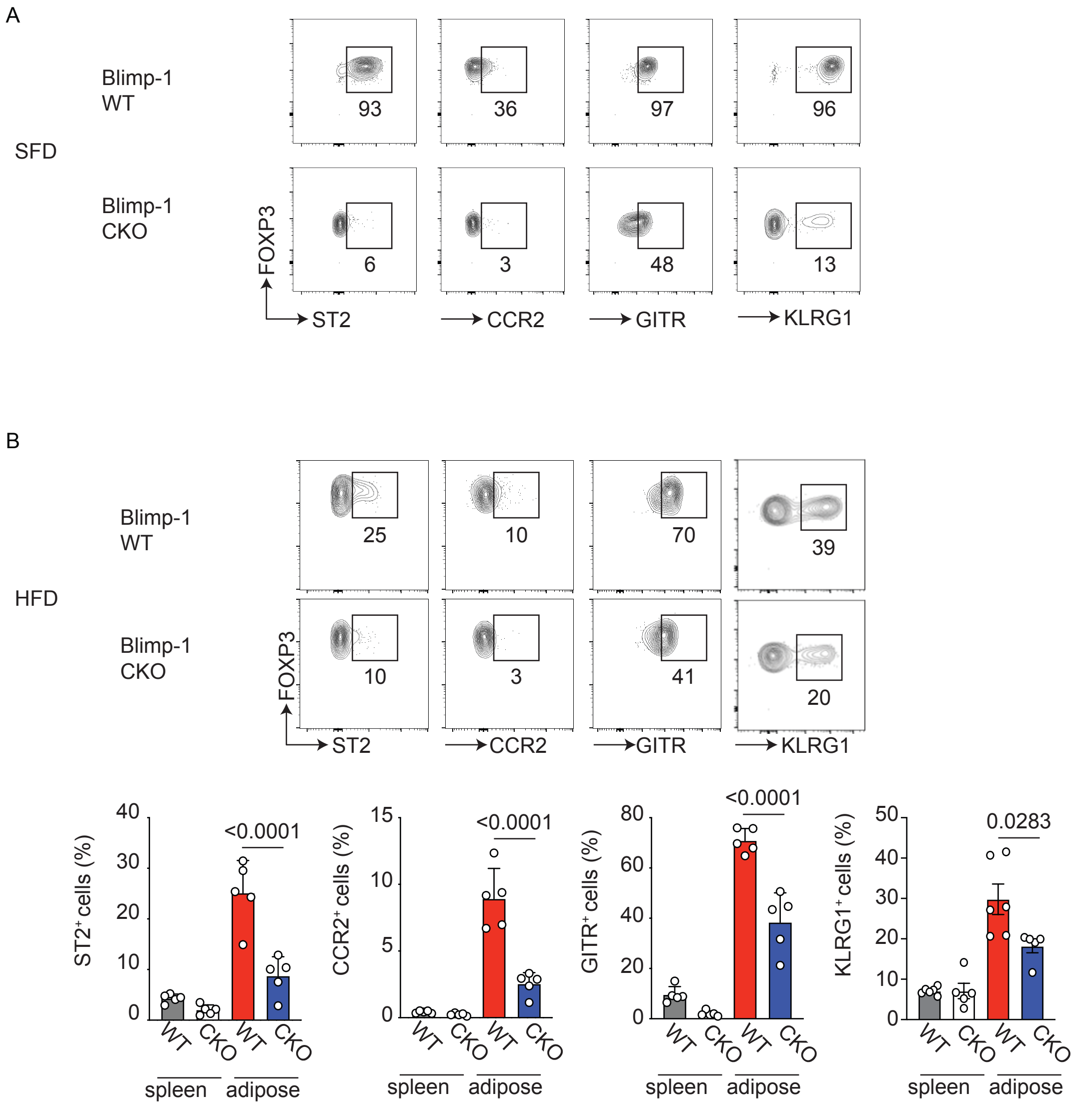

Supplemental Figure 3. Phenotype of Tregs in the VAT with loss of Blimp-1 expression in SFD and HFD-fed mice. Male Foxp3-YFP-Cre+ (WT) and Blimp-1f/f mice crossed to Foxp3-YFP-Cre+ (conditional knockout, CKO) were placed on standard fat diet (SFD) or 60% high fat diet (HFD) at 8 weeks of age for 18-20 weeks prior to analysis. (A) Flow cytometry plots showing ST2, CCR2, GITR and KLRG1 expression on gated CD4+ Foxp3+ Tregs from the VAT of mice on standard fat diet. (B) Flow cytometry and corresponding bar graphs showing ST2, CCR2, GITR and KLRG1 expression on gated CD4+ Foxp3+ Tregs from the spleen and VAT of mice on high fat diet. Data are presented as means ± s.e.m. with n = 5-6 mice per group pooled from two independent experiments. One-way ANOVA was performed to determine significance and the P values are indicated on the graphs.

Supplemental Figure 4

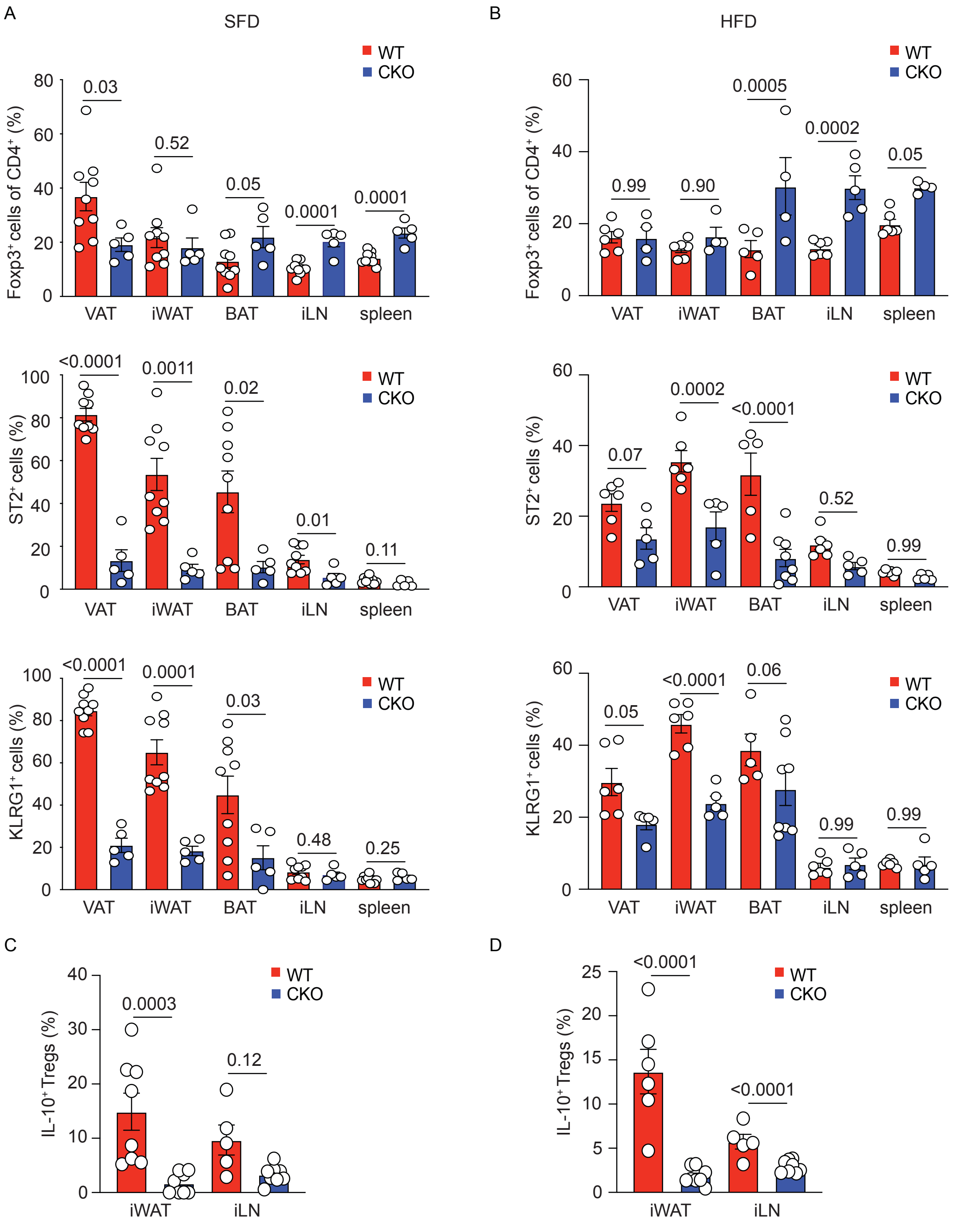

Supplemental Figure 4. Phenotype of Tregs from distinct fat depots with loss of Blimp-1 expression in SFD and HFD-fed mice. Male Foxp3-YFP-Cre+ (WT) and Blimp-1f/f mice crossed to Foxp3-YFP-Cre+ (conditional knockout, CKO) were placed on 20% standard fat diet (SFD) or 60% high fat diet (HFD) at 8 weeks of age for 18-20 weeks prior to analysis. (A) Bar graphs showing the frequency of CD4+ Foxp3+ cells in the visceral adipose tissue (VAT), inguinal white adipose tissue (iWAT), brown adipose tissue (BAT), inguinal lymph nodes (iLN) and spleen from SFD-fed WT and CKO mice. Bar graphs also showing expression of ST2 and KLRG1 on gated CD4+ Foxp3+ cells in the indicated tissue from WT and CKO mice on SFD. (B) Bar graphs showing the frequency of CD4+ Foxp3+ cells in the VAT, iWAT, BAT, iLN and spleen from HFD-fed WT and CKO mice. Bar graphs also showing expression of ST2 and KLRG1 on gated CD4+ Foxp3+ cells in the indicated tissue from WT and CKO mice on HFD. (C) Bar graph showing the percentage of IL-10+ Tregs in gated CD4+ Foxp3+ cells that were stimulated for 5 hours with PMA and ionomycin from the iWAT and iLN of SFD-fed WT and CKO mice. (D) Bar graph showing the percentage of IL-10+ Tregs in gated CD4+ Foxp3+ cells that were stimulated for 5 hours with PMA and ionomycin from the iWAT and iLN of HFD-fed WT and CKO mice. Each dot represents one animal. Data are presented as means ± s.e.m. and are representative of two experiments with 2-5 mice per group for A, B and D, and with 5-8 mice per group for C. One-way ANOVA was performed to determine significance and the P values are indicated on the graphs.

Supplemental Figure 5

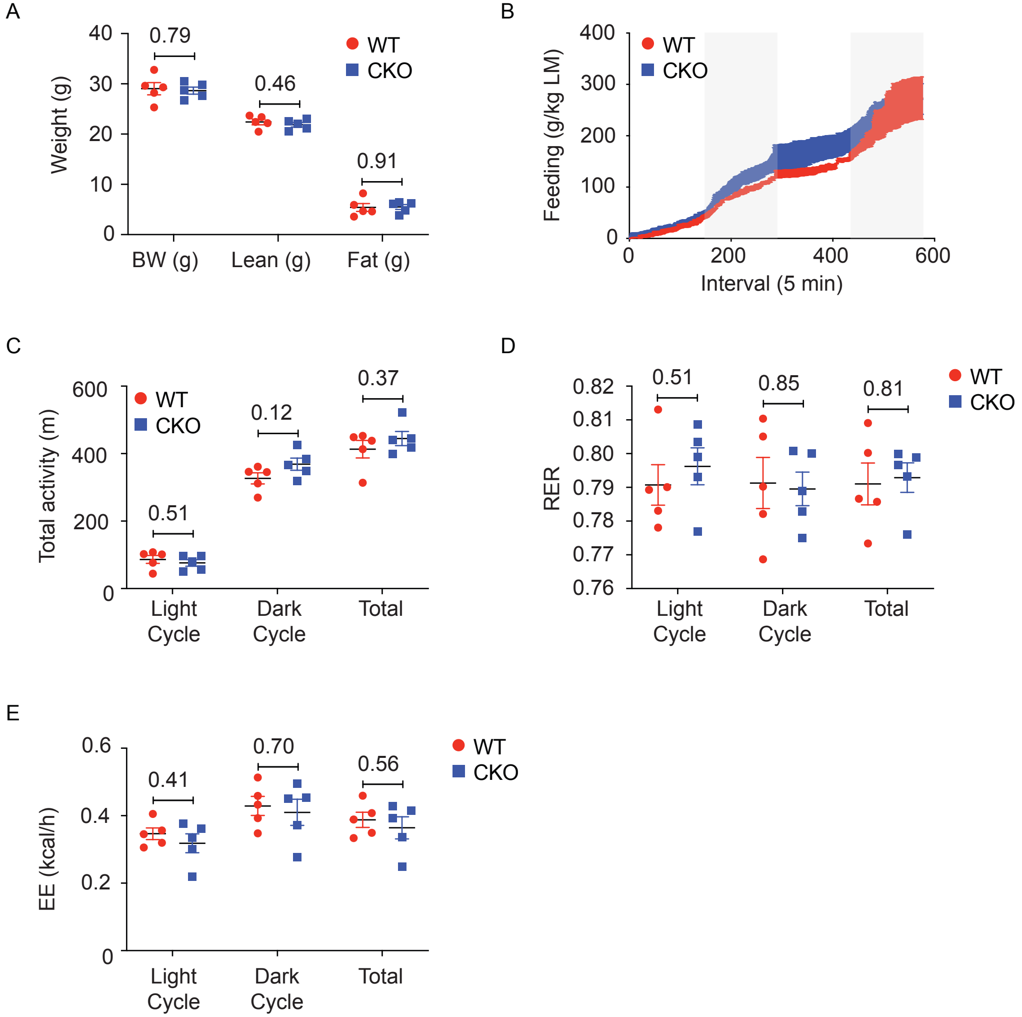

Supplemental Figure 5. Metabolic phenotype of mice on short-term HFD with loss of Blimp-1 expression in Tregs. 8-week-old male Foxp3-YFP-Cre+ (WT) and Blimp-1f/f mice crossed to Foxp3-YFP-Cre+ (conditional knockout, CKO) were placed on 60% high fat diet (HFD) for 3 weeks prior to metabolic analysis. (A) Graph showing body weight (BW), lean and fat mass in grams of WT and CKO mice as measured by EcoMRI. (B-E) Food intake in grams per kilogram lean mass (LM), total activity in meters, respiratory exchange ratio (RER) and energy expenditure (EE) in kcal per hour in light, dark, and total as measured by Promethion Multiplexed Metabolic Cage System during 48-hour total duration. Data are presented as means ± s.e.m. and are representative of two experiments with 2-3 mice per group. An unpaired Student's t-test or one-way ANOVA was performed to determine significance and the P values are indicated on the graphs.

Supplemental Figure 6

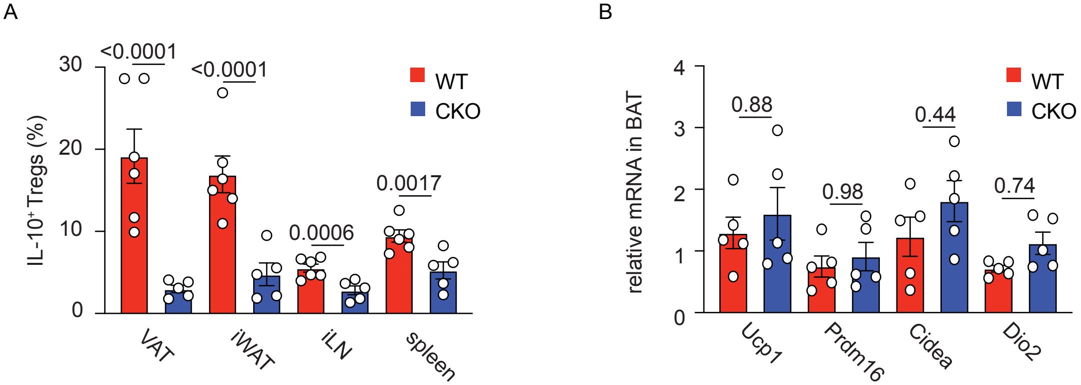

Supplemental Figure 6. Decreased secretion of IL-10 by Blimp-1 deficient aTregs from mice on short-term HFD. 8-week-old male Foxp3-YFP-Cre+ (WT) and Blimp-1f/f mice crossed to Foxp3-YFP-Cre+ (conditional knockout, CKO) were placed on 60% high fat diet (HFD) for 3 weeks prior to analysis. (A) Bar graph showing the percentage of IL-10+ Tregs in gated CD4+ Foxp3+ cells that were stimulated for 5 hours with PMA and ionomycin from the visceral adipose tissue (VAT), inguinal white adipose tissue (iWAT), inguinal lymph node (iLN) and spleen of short-term HFD-fed WT and CKO mice. (B) Bar graph showing relative mRNA expression of the indicated gene of total brown adipose tissue (BAT) from short-term HFD-fed WT and CKO mice. Values were normalized to beta-actin. Each dot represents one animal. Data are presented as means ± s.e.m. and are representative of two experiments with 2-3 mice per group. An unpaired Student’s t-test or one-way ANOVA was performed to determine significance and the P values are indicated on the graphs.

Supplemental Figure 7

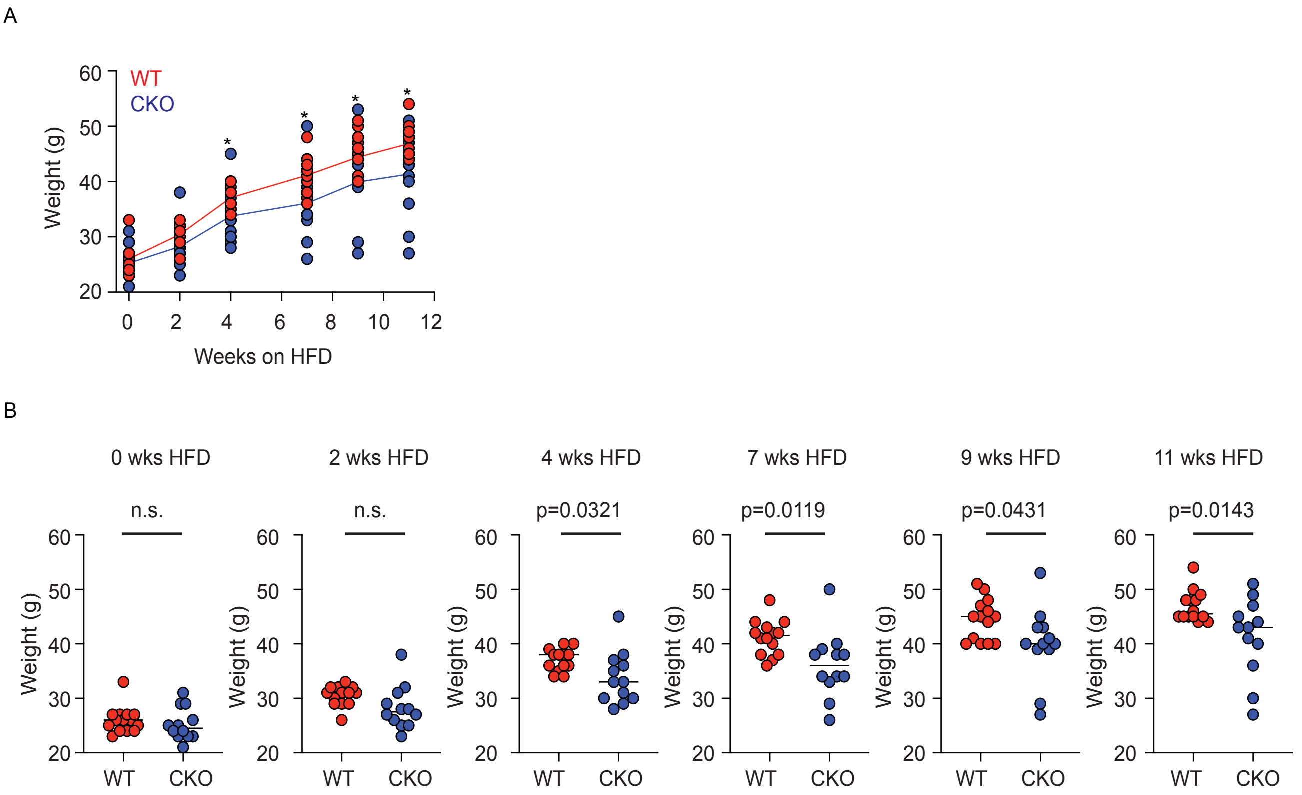

Supplemental Figure 7. Weight gain in WT and Blimp-1-deficient Treg mice on HFD over time. Male Foxp3-YFP-Cre+ (WT) and Blimp-1f/f mice crossed to Foxp3-YFP-Cre+ (conditional knockout, CKO) were placed on 60% high fat diet (HFD) and their weight gain tracked over time. (A) Graph indicating total body weight per mouse per group over time on HFD. (B) Graphs showing the weight in grams of wildtype (WT) or Blimp-1 conditional knockout (CKO) mice on 60% HFD at the time indicated. Data are presented as means ± s.e.m. for n = 12 mice per group pooled from two independent experiments. An unpaired Student’s t-test was performed to determine significance and the P values are indicated on the graphs (n.s. = not significant).

Supplemental Figure 8

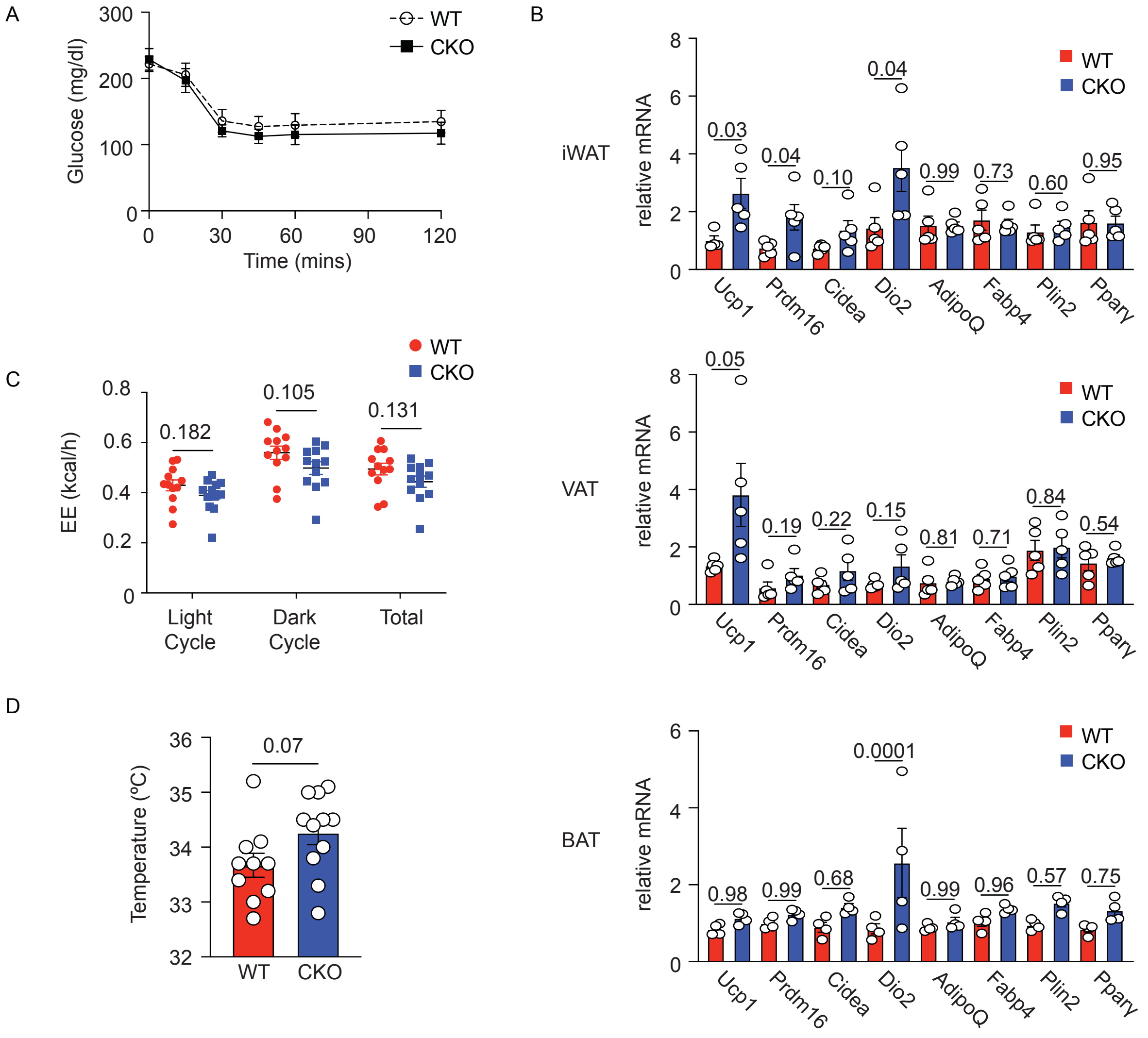

Supplemental Figure 8. Loss of Blimp-1 expression by Tregs increases adipocyte beiging in HFD-fed mice. 8-week-old male Foxp3-YFP-Cre+ (WT) and Blimp-1f/f mice crossed to Foxp3-YFP-Cre+ (conditional knockout, CKO) were placed on 60% high fat diet (HFD) for 18-20 weeks prior to metabolic analysis. (A) An i.p. insulin tolerance test (ITT) was performed on WT and CKO mice. The graph indicates blood glucose levels in mice over time after i.p. insulin injection. (B) Bar graphs showing relative mRNA expression of the indicated gene from total iWAT, VAT and BAT from 26-28-week-old HFD-fed WT and CKO mice. Values were normalized to beta-actin. (C) Graph indicating energy expenditure (EE) in kcal per hour as measured by Promethion Multiplexed Metabolic Cage System during 48-hour total duration. (D) Bar graph indicating rectal temperature in WT and CKO mice. Data are presented as means ± s.e.m., and are from 2-3 independent experiments with 4-12 mice, where each dot represents one mouse and an unpaired Student's t-test, or one-way ANOVA was performed to determine significance.

Supplemental Figure 9

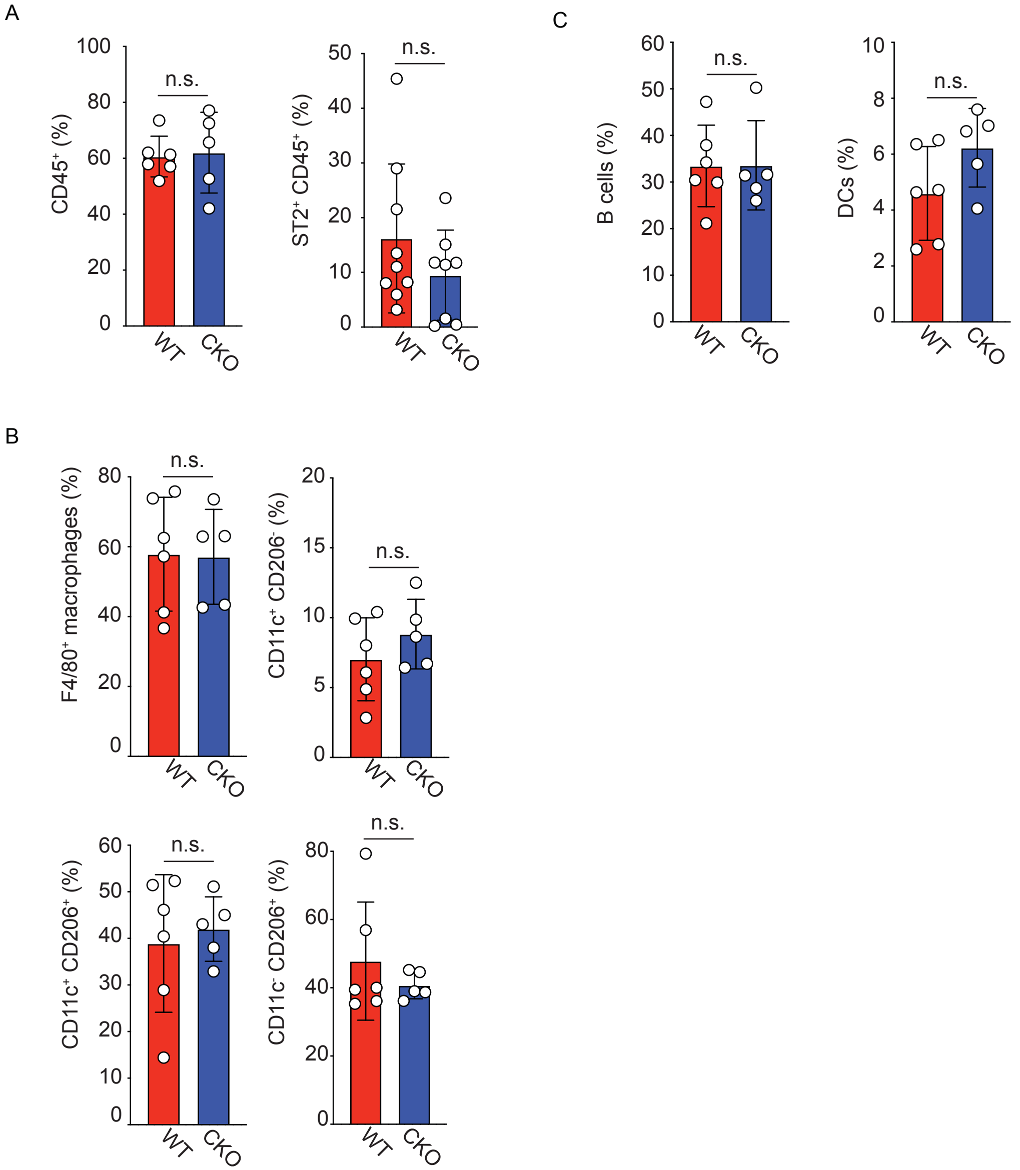

Supplemental Figure 9. Global immune-phenotyping of VAT in Blimp-1 aTreg-deficient animals. 8-week-old male Foxp3-YFP-Cre+ (WT) and Blimp-1f/f mice crossed to Foxp3-YFP-Cre+ (conditional knockout, CKO) were placed on 60% high fat diet (HFD) for 18-20 weeks prior to global immune-phenotyping analysis. (A) Bar graphs indicating the frequency of total CD45+ cells (left) and aTreg- ST2+ CD45+ cells (right) isolated from the stromal vascular fraction (SVF) of WT or CKO mice on HFD. (B) Bar graphs showing the frequency of the indicated macrophage populations isolated from the VAT of WT or CKO mice. (C) Bar graphs showing the frequency of B cells and dendritic cells (DCs) isolated from the VAT of WT or CKO mice. Data are presented as means ± s.e.m., for n = 6-14 mice per group, pooled from at least 2 independent experiments. Each dot represents one mouse and an unpaired Student’s t-test was performed to determine significance.

SUPPLEMENTAL TABLE 1

Gene	Forward (5'-3')	Reverse (5'-3')
Ucp1	GGCCTCTACGACTCAGTCCA	TAAGCCGGCTGAGATCTTGT
Prdm16	CAGCACGGTGAAGCCATTC	GCGTGCATCCGCTTGTG
Cidea	ATCACAACTGGCCTGGTTACG	TACTACCCGGTGTCCATTCT
Dio2	CATGCTGACCTCAGAAGGGC	CCCAGTTTAACCTGTTTGTAGGCA
AdipoQ	GCACTGGCAAGTTCTACTGCAA	GTAGGTGAAGAGAACGGCCTTGT
Fabp4	ACAAGCTGGTGGTGGAATGTG	CCTTTGGCTCATGCCCTTT
Plin2	CTCAGGAGGAGCTGGAGATG	TCAATCAGGTGGACAGTGGA
Pparg	AGGCGAGGGCGATCTTGACAG	AATTCGGATGGCCACCTCTTTG
β-actin	TATTGGCAACGAGCGGTTCC	GGCATAGAGGTCTTTACGGATGT
